# Examining the feasibility of replacing ORF3a with fluorescent genes to construct SARS-CoV-2 reporter viruses

**DOI:** 10.1099/jgv.0.002072

**Published:** 2025-02-12

**Authors:** Isobel Webb, Maximillian Erdmann, Rachel Milligan, Megan Savage, David A. Matthews, Andrew D. Davidson

**Affiliations:** 1 School of Cellular and Molecular Medicine, Faculty of Health and Life Sciences, University of Bristol, Bristol BS8 1TD, UK

**Keywords:** innate immune response, ORF3a, reporter virus, SARS-CoV-2

## Abstract

The SARS-CoV-2 genome encodes at least nine accessory proteins, including innate immune antagonist and putative viroporin ORF3a. ORF3a plays a role in many stages of the viral replication cycle, including immune modulation. We constructed two recombinant (r)SARS-CoV-2 viruses in which the ORF3a gene was replaced with mScarlet (mS) or mNeonGreen (mNG), denoted as rSARS-CoV-2-Δ3a-mS and rSARS-CoV-2-Δ3a-mNG, respectively. rSARS-CoV-2-Δ3a-mNG generated a fluorescent signal after infection in both A549-ACE-2-TMPRSS2 (AAT) and Vero-E6-TMPRSS2 (VTN) cells, unlike rSARS-CoV-2-Δ3a-mS. rSARS-CoV-2-Δ3a-mS mS protein could be detected immunologically in VTN but not AAT cells, indicating the expression of a non-fluorescent mS protein. The analysis of the viral transcriptomes in infected AAT cells by nanopore direct RNA sequencing (dRNAseq) revealed that the level of mS transcript was below the limit of detection in AAT cells. rSARS-CoV-2-Δ3a-mNG virus was found to be genetically stable in AAT and VTN cells, but rSARS-CoV-2-Δ3a-mS acquired partial deletions of the mS gene during sequential passaging in VTN cells, creating the virus rSARS-CoV-2-Δ3a-ΔmS. The mS deletion in VTN cells removes the chromophore coding sequence, and this may explain the presence of a non-fluorescent mS protein detected in VTN cells. The rSARS-CoV-2-Δ3a-mNG, rSARS-CoV-2-Δ3a-mS and rSARS-CoV-2-Δ3a-ΔmS viruses all replicated to a lower titre and produced smaller plaques than the parental rSARS-CoV-2-S-D614G. Interestingly, the rSARS-CoV-2-Δ3a-ΔmS virus produced higher virus titres and larger plaque sizes than rSARS-CoV-2-Δ3a-mS. This suggested that both the insertion of mS coding sequence and the deletion of ORF3a coding sequence contributed to attenuation. In comparison with rSARS-CoV-2, the rSARS-CoV-2-Δ3a-mS and rSARS-CoV-2-Δ3a-mNG viruses showed increased sensitivity to pre-treatment of cells with IFN-α but did not exhibit a dose-dependent increase in replication in the presence of the Janus kinase-signal transducer and activator of transcription signalling pathway inhibitor, ruxolitinib. In conclusion, the replacement of the ORF3a coding sequence with those of fluorescent reporter proteins attenuated the replication of SARS-CoV-2 and its ability to effectively evade the innate immune response *in vitro*.

## Data Availability

The raw reads of the RNA-seq experiment are deposited in the NCBI Sequence Read Archives (SRA) under the BioProject number PRJNA1213676 (accessions SAMN46338597 and SAMN46338598).

## Introduction

Severe acute respiratory syndrome coronavirus 2 (SARS-CoV-2) is the causative agent of the ongoing COVID-19 pandemic [1,3]. It is a *Betacoronavirus* and one of the seven human coronaviruses including SARS-CoV, which cause respiratory tract infections of varying severity [4]. Coronaviruses possess a large, positive-sense ssRNA genome, which encodes non-structural, structural and accessory proteins [5]. The non-structural proteins form the replication–transcription complex (RTC), which mediates genome replication and viral transcription [6]. This RTC structure replicates the full-length genome and transcribes a nested set of sub-genomic RNAs (sgRNAs) from the 3′ end of the genome. The sgRNAs encode the coronavirus structural and accessory proteins and are preceded by transcriptional regulatory sequences (TRSs), which allow for discontinuous transcription [7]. The four structural proteins encoded by SARS-CoV-2 sgRNAs are spike (S), membrane (M), nucleocapsid (N) and envelope (E). At least nine 3′ ORFs in the SARS-CoV-2 genome encode accessory proteins, including ORF3a [5,8]. This work focuses on the largest SARS-CoV-2 accessory protein, ORF3a, which is located between the S and E genes.

ORF3a is a sarbecovirus-specific ORF [8] and was first identified in the SARS-CoV genome after its emergence in 2003 [9,10]. Overlapping the ORF3a sequence are the ORFs encoding the accessory proteins ORF3b [11,12] and ORF3c [13]. The SARS-CoV and SARS-CoV-2 ORF3a proteins share 73% aa sequence homology and have both been proposed as putative cation-selective ion channels, which are embedded into cellular membranes [14,16]. A recent report investigating the SARS-CoV and SARS-CoV-2 ORF3a proteins states that neither protein is capable of forming functional pores [17]. The ORF3a protein possesses a PDZ-binding motif (PBM) domain at its C-terminus [18,19], allowing for binding to cellular proteins with a PDZ domain and consequently regulation of signalling complexes [20]. It has been reported that 270 cellular proteins possess a PBM [21] and therefore possession of this domain potentially allows for wide-scale alteration of the cellular environment.

The ORF3a protein is implicated in various viral processes, including viral entry, replication, transcription, immune regulation, pathogenesis, assembly and release [14,15, 22], as reviewed [23]. Additionally, ORF3a overexpression studies have identified a role in apoptosis and necrosis [24,25]. The ORF3a protein has been shown to play a critical role in viral pathogenesis, as demonstrated by an *in vivo* study where mice infected with a recombinant SARS-CoV lacking ORF3a exhibited 100% survival, in contrast to mice infected with the parental virus which did not survive [19]. Deletion of the ORF3a coding sequence was previously shown not to impact viral replication *in vitro* [26].

SARS-CoV-2 induction of a pro-inflammatory signalling overreaction is indicative of clinical outcome, with patients experiencing a cytokine storm at higher risk of serious disease and death [27]. Type I (*α*/*β*) and III (*λ*) IFNs are secreted cytokines, which are produced upon virus infection [28]. Secreted IFNs are detected by neighbouring cells, via the Janus kinase (JAK)-signal transducer and activator of transcription (STAT) signalling pathway, to prime their antiviral response and reduce susceptibility to subsequent viral infection [29]. SARS-CoV-2 replication is impaired in the presence of type I IFNs, and a number of SARS-CoV-2 proteins modulate IFN secretion and JAK-STAT signalling to promote viral infection and pathogenesis [30,33].

The ORF3a protein triggers a range of innate immune responses, including cell stress and pro-inflammatory responses. Upon exogenous expression, it has been shown to activate the pro-inflammatory response, resulting in the production of several cytokines, likely mediated through NFκB [34]. This activation can initiate a cytokine storm via the activation of the inflammasome in infected cells [35]. Additionally, ORF3a blocks type I/III IFN signalling through the JAK-STAT pathway by upregulating suppressor of cytokine signalling 1 (SOCS1), degradation of Janus kinase 2 (JAK2) [36] and antagonism of STAT1 phosphorylation [37].

Recombinant SARS-CoV-2 viruses expressing reporter genes are valuable tools to track viral infection and screen potential antiviral compounds. Previous studies have replaced various accessory genes in the SARS-CoV-2 genome with fluorescent reporters to generate reporter viruses. This strategy has largely involved the insertion of a fluorescent reporter gene in the ORF7a coding sequence [38,40]. ORF6 and ORF8 have also been replaced [41]. Deletion of the ORF3a gene has been reported not to result in an attenuated replication phenotype *in vitro*, unlike deletion of ORF7a [26]. Therefore, recombinant (r)SARS-CoV-2 viruses were generated in which the ORF3a coding sequence was partially replaced with the coding sequences of either mScarlet (mS) [42] (rSARS-CoV-2-Δ3a-mS) or mNeonGreen (mNG) [43] (rSARS-CoV-2-Δ3a-mNG) to investigate the role of ORF3a in virus replication and to determine the suitability of ORF3a replacement for generating fluorescent reporter viruses. This study demonstrated that the feasibility of replacing ORF3a for the generation of reporter viruses was dependent on both the fluorescent reporter and cell type used. Ultimately, rSARS-CoV-2-Δ3a-mNG, unlike rSARS-CoV-2-Δ3a-mS, was found to be a useful reporter virus.

## Methods

### Cells

Human A549 cells modified to stably express angiotensin-converting enzyme 2 (ACE2) and transmembrane protease, serine 2 (TMPRSS2; termed AAT cells), African green monkey kidney (Vero-E6; ATCC^®^ CRL 1586TM) cells stably expressing both ACE2 and TMPRSS2 (termed VAT cells) and baby hamster kidney (BHK-21) cells stably expressing ACE2 and the SARS-CoV-2 N protein (termed BAN cells) were a kind gift from Professor Arvind Patel and Dr Suzannah Rihn, Centre for Virus Research, University of Glasgow [44]. Vero-E6 cells modified to stably express TMPRSS2 (termed VTN cells) were sourced from the National Institute for Biological Standards and Control (Hertfordshire, UK). Human bronchial epithelium cells BEAS-2B modified to stably express ACE2 (termed BEAS-2BA cells) were kindly provided by Professor Stuart Neil and Dr Harry Wilson, King’s College London. All cell lines were cultured at 37 °C with 5% CO_2_ in cell growth media consisting of Dulbecco’s modified Eagle’s medium (Gibco™, ThermoFisher) supplemented with 0.1 mM minimal essential medium (MEM) non-essential aa (Gibco™, ThermoFisher) and 10% (v/v) FBS (Gibco™, ThermoFisher). Cell infection media consisted of Eagle’s MEM (Gibco™, ThermoFisher) supplemented with 0.1 mM MEM non-essential aa and 2% (v/v) FBS.

DH10B *Escherichia coli* (ECo113, ThermoFisher) were used to amplify SARS-CoV-2 cDNA fragments in the vector pJET1.2/blunt (ThermoFisher) and grown on lysogeny broth (LB) agar plates with ampicillin (100 µg ml^−1^) at 37 °C overnight. OneShot^®^ Top10 Electrocomp™ *E. coli* (Invitrogen™, ThermoFisher) were used to propagate the GeneArt™ pYES1L vector (Invitrogen™, ThermoFisher) containing full-length SARS-CoV-2 cDNA clones and were grown overnight at 37 °C on LB agar or shaking in LB supplemented with spectinomycin at 100 µg ml^−1^ and 50 µg ml^−1^, respectively.

MaV203 competent yeast cells (*MATα*, *leu2-3,112*, *trp1-901*, *his3Δ200*, *ade2-101*, *cyh2^R^*, *can1^R^*, *gal4Δ*, *gal80Δ*, *GAL1::lacZ*, *HIS3_UASGAL1_::HIS3@LYS2* and *SPAL10_UASGAL1_::URA3*; Invitrogen™, ThermoFisher) were used for yeast-based transformation-associated recombination (TAR) and propagation of inserts contained in the pYES1L vector. Yeast was grown on 2% (w/v) agar plates containing yeast nitrogen base without aa (6.8 g l^−1^, Sigma-Aldrich) supplemented with yeast synthetic dropout medium supplement without tryptophan (1.92 g l^−1^; Sigma-Aldrich) and 2% (w/v) d-(+)-glucose (YSM-Trp plates) at 30 °C.

### Construction of rSARS-CoV-2 with the ORF3a coding sequence partially replaced with the mS and mNG coding sequences

In order to produce a genome length SARS-CoV-2 cDNA clone, 11 cDNA fragments with 70 bp end-terminal overlaps that spanned the entire SARS-CoV-2 isolate Wuhan-Hu-1 genome (GenBank accession: NC_045512) were produced by GeneArt™ synthesis (Invitrogen™, ThermoFisher) as cDNA inserts in sequence-verified, stable plasmid clones. The 5′ terminal cDNA fragment was modified to contain 70 nt corresponding to nt 9311–9380 of the pYES1L vector, a T7 RNA polymerase promoter and an extra ‘G’ nt immediately upstream of the SARS-CoV-2 5′ genome sequence, whilst the 3′ terminal cDNA fragment was modified such that the 3′ end of the SARS-CoV-2 genome was followed by a stretch of 33 ‘A’s followed by the unique restriction enzyme site *Asc*I and 70 nt corresponding to nt 1–70 of the pYESL1 vector. The SARS-CoV-2 cDNA fragment contained in each clone was PCR amplified using gene-specific primer pairs and the Platinum™ SuperFi II PCR Master Mix (Invitrogen™, ThermoFisher) following the manufacturer’s instructions, confirmed by DNA gel electrophoresis and purified using a GeneJET Gel extraction kit (ThermoFisher) following the manufacturer’s instructions.

From the original SARS-CoV-2 cDNA clones, clones were prepared that were designed to replace the individual genes ORF3a and ORF6 with genes encoding either of the fluorescent reporter proteins mNG or mS. The sequences of mNG [43] and mS [42] were codon optimized for the expression in human cells and introduced into plasmids as sub-genomic SARS-CoV-2 cDNA fragments by GeneArt™ synthesis. The three resulting viruses were denoted as rSARS-CoV-2-Δ3a-mNG, rSARS-CoV-2-Δ3a-mS and rSARS-CoV-2-Δ6-mS. The rSARS-CoV-2-Δ3a-NG and rSARS-CoV-2-Δ3a-mS viruses contain a partial deletion of the ORF3a coding sequence (relative to Wuhan-Hu-1 isolate, GenBank accession: NC_045512) from nt 25393–25752 and sub-genomic replacement with codon-optimized mNG and mS. The rSARS-CoV-2-Δ6-mS virus contains a sub-genomic replacement of ORF6 coding sequence (nt 27202–27371) with codon-optimized mS. The vectors containing mNG and mS in place of the ORF3a or ORF6 genes were cleaved with the same enzymes, respectively, and the appropriate DNA fragments were gel purified and cloned into the pJET1.2–1011 backbones. The resulting clones were sequence verified, and fragments were amplified from the clones by PCR for TAR assembly.

SARS-CoV-2 cDNA fragments were assembled into full-length clones by TAR following a previously established method [39] using the GeneArt™ High-Order Genetic Assembly System (Invitrogen, ThermoFisher) according to the manufacturer’s instructions. Briefly, 200 ng of each fragment was added to a tube with 100 ng of the GeneArt® pYES1L Vector with Sapphire™ Technology, and the volume was reduced to 5–10 µl using a SpeedVac. One hundred microlitres of thawed MaV203 competent yeast cells were added to the tube, followed by 600 µl of polyethylene glycol / lithium acetate. The solutions were mixed gently and incubated at 30 °C for 30 min, followed by the addition of 35.5 µl of molecular grade DMSO (Sigma-Aldrich), further mixing and incubation at 42 °C for 20 min. The yeast was then plated on YSM-Trp plates and grown at 30 °C until colonies were visible. DNA was extracted from yeast clones following the method described in the GeneArt™ High-Order Genetic Assembly System, and the clones were screened for successful assembly by multiplex PCR using the Platinum™ SuperFi II PCR Master Mix and 2 sets of primer pairs collectively targeting all 11 recombinant junctions, both in the SARS-CoV-2 genome and the pYES1L vector. All primer pairs produce products of distinct sizes, which were confirmed by agarose gel electrophoresis [45]. pYES1L clones containing correctly assembled SARS-CoV-2 cDNA clones were electroporated into OneShot™ Top10 Electrocomp™ *E. coli* and grown at 37 °C overnight under spectinomycin selection. Colonies were picked and amplified by large shaking culture in LB containing spectinomycin at 37 °C overnight before being purified using NucleoBond™ Xtra BAC, a large construct maxi-prep according to the manufacturer’s instructions (Macherey-Nagel, Fisher Scientific).

The SARS-CoV-2 cDNA clones contained in pYES1L were linearized by restriction digest with *Asc*I (New England BioLabs, Inc., Ipswich, MA) and purified by phenol-chloroform/chloroform extraction followed by salt-ethanol precipitation. The precipitated DNA was washed three times in ultra-pure 75% (v/v) ethanol and resuspended in nuclease-free water. *In vitro* transcription (IVT) of RNA from template DNA (1.5 µg/reaction) was performed in a 50 µl volume using the RiboMAX™ Large Scale RNA Production System-T7 (Promega) following the manufacturer’s instructions for 3 h at 30 °C except that the concentration of GTP was reduced to 1.5 mM and the cap structure analogue m7G(5′)ppp(5′)G RNA (Promega) was added to a final concentration of 3 mM. The provided RQ1 RNase-Free DNase was then added to 1 U µg^−1^ template DNA, and the mix was incubated at 37 °C for a further 15 min. RNA was then immediately purified by lithium chloride precipitation using LiCl Precipitation Solution (7.5 M) (Invitrogen™) following the manufacturer’s recommended protocol and re-suspended in nuclease-free water; or when used downstream as the IVT reaction mix, the reaction was immediately stored at –70 °C. Aliquots of purified RNA or IVT were checked for integrity by denaturing agarose-formaldehyde gel electrophoresis.

*In vitro* RNA transcripts were transfected into cells either before or after purification using the Neon® Transfection System as per the manufacturer’s instructions (ThermoFisher). Purified *in vitro* RNA transcripts (10 µg of genomic transcripts and 2 µg of N gene transcript when used) were prepared in 10 µl of nuclease-free water. Alternatively, either 5 or 10 µl from a 50 µl IVT reaction (and 2 µg of N gene transcript when used) was used directly for transfection. Cells were trypsinized and washed in Dulbecco’s phosphate-buffered saline before resuspension in resuspension buffer R at a density of 1×10^7^ cells per millilitre. For each transfection, 100 µl of cells was mixed with RNA/transcription mix (maximum volume 10 µl). Cells were then transfected using a Neon™ Transfection System (Invitrogen™) with the following electroporation settings for BAN (1200 V, 30 ms, 1 pulse) and VTN cells (1150 V, 20 ms, 2 pulses). Cells were immediately resuspended in warm media and seeded into a cell culture flask. Cells were incubated at 37 °C in 5% CO_2_ until viral cytopathic effects (CPEs) were observed. Supernatants were then transferred onto fresh VTN cells with the addition of fresh media and incubated as before until viral CPE was again observed. Supernatants were filtered through a 0.2 µM filter, buffered to 25 mM HEPES (pH 7.0) and frozen at −80 °C as the infectious viral stock. Viral stocks used in this study are at passage 1 (p1) and were propagated in VAT cells with the supernatant harvested at 72 h post-infection (hpi).

### SARS-CoV-2 infection assays

Cells were seeded the day prior to infection in appropriate media and culture vessels. For infection, the culture supernatants were removed, and the cells were incubated with the relevant virus diluted in the infection medium. After an appropriate time, the inoculum was removed, and the cells were washed once with infection medium. The cells were then incubated in the infection medium at 37 °C in 5% CO_2_ until required for further analysis. All work with infectious SARS-CoV-2 was done inside a class III microbiological safety cabinet in a containment level 3 facility at the University of Bristol and approved by the UK Health and Safety Executive.

### Full genome sequence analysis of recombinant SARS-CoV-2 viruses

RNA was extracted from p1 viral stocks using a QIAamp^®^ RNA Mini Kit (Qiagen) following the manufacturer’s instructions. Illumina mRNA sequencing was performed at the Genomics Facility, University of Bristol, using the NEBNEXT^®^ Artic SARS-CoV-2 Library Prep Kit (New England BioLabs, Inc.) followed by sequencing on an Illumina MiSeq instrument using an Illumina version 3, 600-cycle sequencing kit (Illumina Inc., San Diego, CA) and MiSeq Control Software version 2.5.0.5. Primary data analysis was performed using MiSeq Software (RTA version 1.18.54.0) and FASTQ Creator (MiSeq Reporter version 2.5.1.3) followed by downstream analysis using bespoke scripts developed in-house.

### Quantitative immunofluorescence assay to determine viral infectivity and fluorescent reporter protein expression by automated image analysis

AAT or VTN cells seeded into µClear 96-well Microplates (Greiner Bio-One) were infected with twofold serial dilutions (up to 1:256) of recombinant virus stocks in the infection medium. To determine either viral infectivity or reporter protein fluorescence, cells were fixed at appropriate times post-infection with a final concentration of 4% (v/v) paraformaldehyde (PFA) in PBS for 1 h at room temperature. Following fixation, cells were washed once in PBS and permeabilized with 0.1% (v/v) Triton X-100 in PBS for 10 min at room temperature. Cells were washed once in PBS and then blocked for 1 h in 1% (w/v) BSA in PBS. Cells were incubated for 45 min with a primary antibody against the SARS-CoV-2 N protein (1:2000; 200-401-A50, Rockland) or mS (anti-mCherry antibody 1:300; ab167453, Abcam). mS was derived from a synthetic sequence of the monomeric red fluorescent protein (RFP) [42] and retains antigenicity to an mCherry RFP antibody [46]; for clarity, the anti-mCherry antibody will be referred to as anti-mS. The primary antibody was removed from cells prior to three washes in PBS. Cells were incubated for 1 h with a secondary antibody (Alexa Fluor anti-rabbit 647; 1:3000; Invitrogen™, ThermoFisher) and the nuclear stain Hoechst 33342 (1:10000, Merck). To determine the number of virus-infected cells/cells expressing mS/mNG, images were acquired using an ImageXpress Pico Automated Cell Imaging System (Molecular Devices, Wokingham, UK) using a 10× objective. The number of N protein/mS/mNG-positive cells was quantified using the CellReporterXpress software (Molecular Devices). For the calculation of m.o.i. values, virus stocks were titred on VTN cells in 96-well plates. Unless stated otherwise, the cells were fixed at 6 hpi, and the number of virus-infected cells was determined as described above using an antibody against the viral N protein and nuclear staining with Hoechst 3342. The virus titre was then calculated as virus infectious units per millilitre (IU/ml).

### Viral transcriptomic analysis

Recombinant viruses were used to infect AAT cells at an m.o.i. of 0.1 (IU/cell). At 24 hpi, total RNA was extracted from the cell sheet using TRIzol reagent (ThermoFisher) prior to RNA sequencing using a Direct RNA Sequencing Kit and Oxford Nanopore flow cell (Oxford Nanopore Technologies, Oxford, UK) as described previously [47]. The sequenced reads were aligned to the SARS-CoV-2 genome (GenBank accession number NC_045512) or the modified rSARS-CoV-2-Δ3a-mS and rSARS-CoV-2-Δ3a-mNG sequences using an ORF-centric pipeline developed at the University of Bristol using minimap2 [47,48]. The total number of sgRNA transcripts mapping to the viral genome using the ORF-centric pipeline was determined, and relative amounts of each sgRNA were expressed as a per cent of the total.

### Determination of the genetic stability of recombinant viruses by serial passage *in vitro*

Confluent AAT or VTN cell monolayers in 6-well plates were infected with recombinant viruses at an m.o.i. of 0.01 (IU/cell). At 2 hpi, the inoculum was removed, and the cells were washed and incubated further in the infection medium for 72 h. The culture supernatants were harvested, alongside media from mock-infected wells and diluted one in ten for subsequent inoculation. The process was repeated up to p5. RNA was extracted from the passaged viral stocks using a QIAamp® RNA Mini Kit following the manufacturer’s instructions. A 3197 bp region spanning the 3′ terminal 1292 nt of the S sequence to nt 88 of the ORF6 sequence and encompassing the ORF3a modifications (nt 24093–27289; relative to GenBank accession number NC_045512) was amplified by reverse transcriptase-polymerase chain reaction (RT-PCR) and Sanger sequenced by Eurofins Genomics (Ebersberg, Germany) using primers 5′-TTGCTGCTAGAGACCTCATTTG-3′ (nt 24093–24114) and 5′-CAAGATTCCAAATGGAA ACTTTAAAAGTCC-3′ (nt 27260–27289).

### Analysis of viral replication kinetics *in vitro*

AAT, BEAS-2BA or VTN cells, seeded into 24-well plates, were infected with recombinant viruses at an m.o.i. of 0.01 (IU/cell). At 2 hpi, the viral inoculum was removed, and the cells were washed with infection media followed by the addition of 1 ml of infection media. Three independent experiments were performed in which culture supernatants were sampled and replaced at 4, 8, 24, 48 and 72 hpi, and the infectious virus was quantified in triplicate using quantitative immunofluorescence assay (IFA), as described in the section ‘Quantitative immunofluorescence assay to determine viral infectivity and fluorescent reporter protein expression by automated image analysis’.

### Quantification of plaque size

Confluent VTN cell monolayers in 6-well plates were inoculated with 500 µl of tenfold serial dilutions of virus in infection media. At 1 hpi, the virus was removed, and the cells were washed with infection medium and overlayed with infection media containing 1.25% (w/v) Avicel (FMC BioPolymer). Three days post-infection cells were fixed with 4% (v/v) formaldehyde and stained with 0.1% crystal violet (Sigma-Aldrich). Plaque assays were performed in biological triplicate per virus, and plaque sizes were measured from 10 plaques per biological repeat using NIH ImageJ software [49], totalling 30 plaques per virus.

### IFN dose-response assay

AAT or VTN cells grown in 24-well plates were pre-treated with titrated amounts of type I IFN [IFN-*α*; universal type I IFN (human IFN-alpha hybrid protein, 11200-1-PBL, Stratech, Piscataway, NJ, USA] for 18 h. Cells were washed once with infection media to remove IFN and infected with viruses at an m.o.i. of 0.01 (IU/cell). At 2 hpi, the virus inoculum was removed, and the cells were washed and incubated further in the infection medium. At 24 hpi (VTN) or 48 hpi (AAT), the culture supernatants were harvested, and the infectious virus was quantified in triplicate using IFA, as described in the section ‘Quantitative immunofluorescence assay to determine viral infectivity and fluorescent reporter protein expression by automated image analysis’. Three independent biological repeats were performed for this assay.

### Ruxolitinib dose-response assay

AAT cells grown to confluency in a 96-well plate were pre-incubated with titrated amounts of ruxolitinib (SM87-2, Cell Guidance Systems, St. Louis, MO, USA) for 2 h, prior to infection. Cells were inoculated with viruses at an m.o.i. of 0.025 (IU/cell). At 2 hpi, the inoculum was removed, and the cells were washed and incubated further in the infection medium containing a twofold dilution series of ruxolitinib (ranging from 0.3 to 5 µM). At 24 hpi, the cells were fixed, and the per cent of N protein-positive cells was determined using IFA, as described in the section ‘Quantitative immunofluorescence assay to determine viral infectivity and fluorescent reporter protein expression by automated image analysis’. Three independent biological repeats were performed for this assay.

### Quantification of host gene transcripts by real-time quantitative PCR

AAT cells grown to confluency in 12-well plates were pre-incubated with 5 µM ruxolitinib for 2 h, prior to infection. Cells were inoculated with viruses at an m.o.i. of 0.01 (IU/cell) or mock infected. At 2 hpi, the inoculum was removed, and the cells were washed and incubated further in the infection medium containing 5 µM ruxolitinib. At 24 hpi, total RNA was extracted from the cells using an SV Total RNA Isolation System (Promega) following the manufacturer’s protocol. Total RNA was reverse transcribed using a GoScript Reverse Transcription System (Promega). The resulting cDNA was diluted so that 250 ng was added per qPCR reaction. For cellular gene transcripts, qPCR was performed using the Fast SYBR™ Green Master Mix (ThermoFisher). Host gene transcript primer sequences were obtained from work published previously [50], and the expression was determined using the 2^−ΔΔCt^ method [51] and normalized to the GAPDH transcript. Quantification of SARS-CoV-2 genome copy numbers by real-time quantitative PCR (RT-qPCR) was done as previously described [52] using the TaqPath™ 1-Step RT-qPCR Master Mix (ThermoFisher). All reactions were run on a BioRad CFX Opus 96 Real-Time PCR System.

### Statistical analysis

Statistical analysis was performed using GraphPad Prism version 10. The standard deviation and normality were assessed before any statistical analysis. Three independent biological repeats were performed for each experiment, and significance was taken as *P*-value <0.05.

## Results

### Construction of rSARS-CoV-2 viruses with the ORF3a coding sequence replaced by those of mNG or mS

**Fig. 1.**
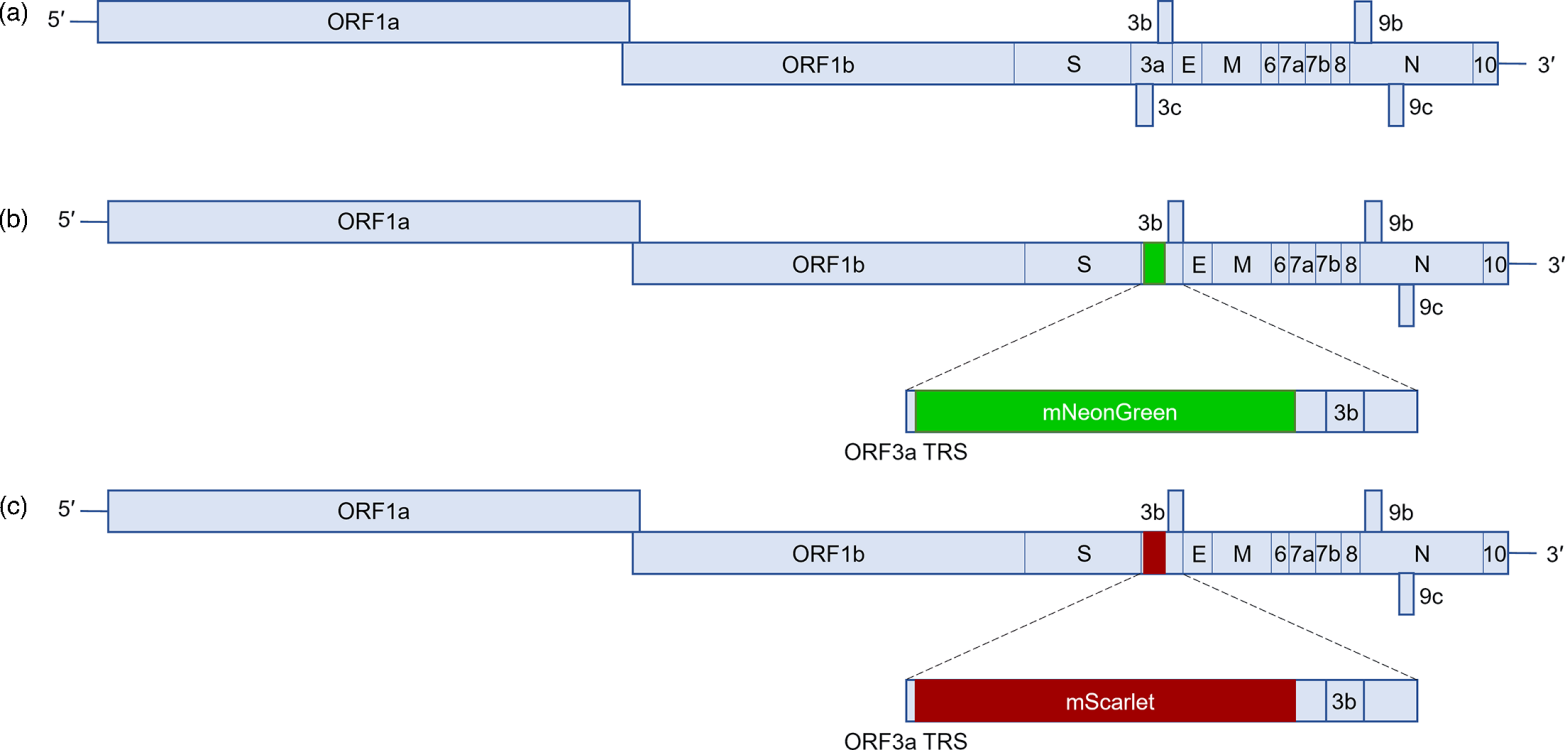
Genome organization of rSARS-CoV-2, rSARS-CoV-2-Δ3a-mNG and rSARS-CoV-2-Δ3a-mS. (**a**) Schematic of the rSARS-CoV-2 genome and the recombinant viruses (**b**) rSARS-CoV-2-Δ3a-mNG and (**c**) rSARS-CoV-2-Δ3a-mS generated in this study. The position of the ORF3a gene is highlighted either in rSARS-CoV-2 (**a**) or with the fluorescent protein gene reporters (**b, c**).

The ORF3a coding sequence contains the coding sequences of both the ORF3b and ORF3c accessory proteins (Fig. 1a). For this study, recombinant viruses were constructed in which the ORF3a coding sequence (nt 25393–26220) was partially replaced (nt 25393–25752) with the coding sequences of either mNG (711 nt) [43] or mS (699 nt) [42], each terminated by an in-frame translation stop codon and denoted rSARS-CoV-2-Δ3a-mNG (Fig. 1b) and rSARS-CoV-2-Δ3a-mS (Fig. 1c), respectively. Retention of the ORF3a 3′ terminal 468 nt (nt 25753–26220) in the constructs ensured that the ORF3b coding sequence [11] was undisturbed (Fig. 1b, c). By contrast, the ORF3c coding sequence [13,53] was deleted during the cloning process (Fig. 1). A previously described, yeast-based reverse genetic system based on the Wuhan-Hu-1 isolate reference sequence (GenBank accession number NC_045512) [39,45] was used to generate full-length cDNA clones (in a YAC/BAC vector) corresponding to the rSARS-CoV-2 viruses. The A23403G mutation in the S gene, resulting in the D614G substitution, quickly became prevalent in SARS-CoV-2 sequences [54,55] and was therefore included within these constructs. A control virus denoted as rSARS-CoV-2 was generated using the same reverse genetic system and contained the additional D614G mutation in the S gene.

The recombinant viruses were rescued from the YAC/BAC clones by transfection of T7 polymerase generated *in vitro* SARS-CoV-2 RNA transcripts into BAN cells. Working stocks (p1) of the viruses were generated by inoculation of VAT cells with culture supernatants from the transfected BAN cells. The viruses in the p1 stocks were then sequenced by Illumina amplicon-based sequencing. As the mNG and mS insertions caused a dropout of specific amplicons, the sequences of the corresponding genomic regions were amplified by RT-PCR and confirmed by Sanger sequencing. For both rSARS-CoV-2-Δ3a-NG and rSARS-CoV-2-Δ3a-mS, the consensus sequences generated confirmed the presence of the mNG or mS replacement in the desired region. The rSARS-CoV-2-Δ3a-mS virus had an additional G17259T mutation at 70% read frequency, which was not present in the progenitor cDNA clone. The mutation resulted in a conservative E341D aa change in the nsp13 protein. No mutations were found in other regions in the genome of either the rSARS-CoV-2-Δ3a-mNG or rSARS-CoV-2-Δ3a-mS viruses. The sequenced p1 viruses were used for further experiments.

### rSARS-CoV-2-Δ3a-mNG expresses a functional fluorescent protein unlike rSARS-CoV-2-Δ3a-mS

**Fig. 2.**
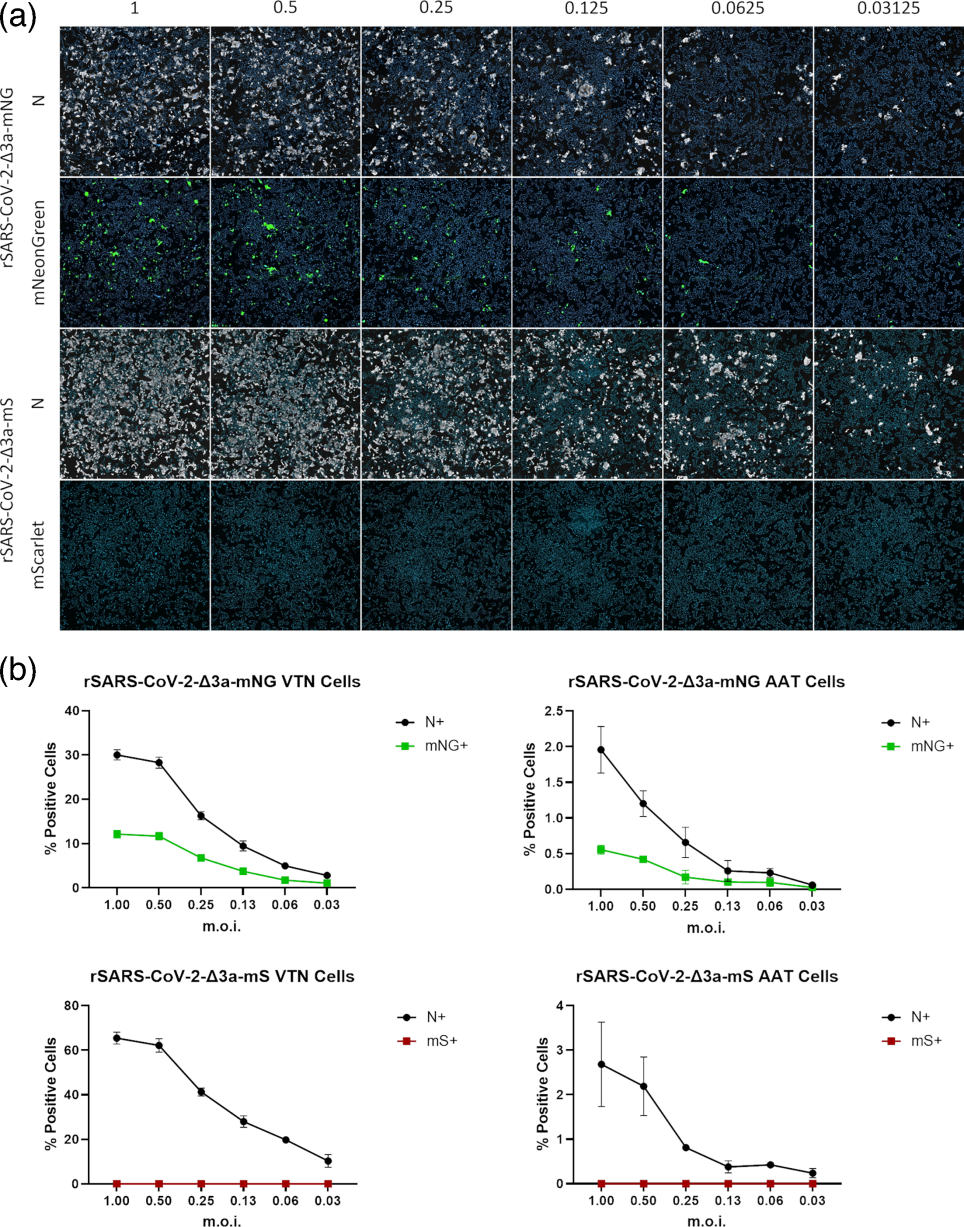
Expression of reporter proteins by rSARS-CoV-2-Δ3a-mNG and rSARS-CoV-2-Δ3a-mS. VTN or AAT cells were inoculated with either rSARS-CoV-2-Δ3a-mNG or rSARS-CoV-2-Δ3a-mS at the indicated m.o.i. values [IU/cell; virus titre (IU/ml) determined at 18 hpi] and incubated for 18 h. (**a**) VTN cells were fixed in 4% PFA at 24 hpi and permeabilized with 0.1% Triton. Cells were stained for SARS-CoV-2 N protein, and nuclei were counterstained with Hoechst. (**b**) VTN and AAT cells were imaged using an ImageXpress Pico Automated Cell Imaging System, and the per cent of cells positive for either N protein (N+), mNG (mNG+) or mS (mS+) was determined using the CellReporterXpress software. Graphs show the mean and sem of *N*=3 repeats.

To establish whether the expression of the mNG and mS reporter proteins could be used to faithfully identify cells infected by the respective viruses, fluorescence from the reporter proteins was assessed in comparison with the detection of the SARS-CoV-2 N protein by IFA. VTN and AAT cells were infected with rSARS-CoV-2-Δ3a-mNG or rSARS-CoV-2-Δ3a-mS at a range of m.o.i. values. At 18 hpi, the cells were fixed, and the number of N protein-positive and fluorescent cells was determined (Fig. 2). Across the different m.o.i. values, ~50% of VTN and 25% of AAT cells found to be infected with rSARS-CoV-2-Δ3a-mNG (assessed by N protein staining) were fluorescent at the time of fixation. Although rSARS-CoV-2-Δ3a-mS infection was readily detectable by N protein staining, no fluorescence could be quantified above background in either VTN or AAT cells.

### rSARS-CoV-2-Δ3a-mS produces non-fluorescent mS protein

**Fig. 3.**
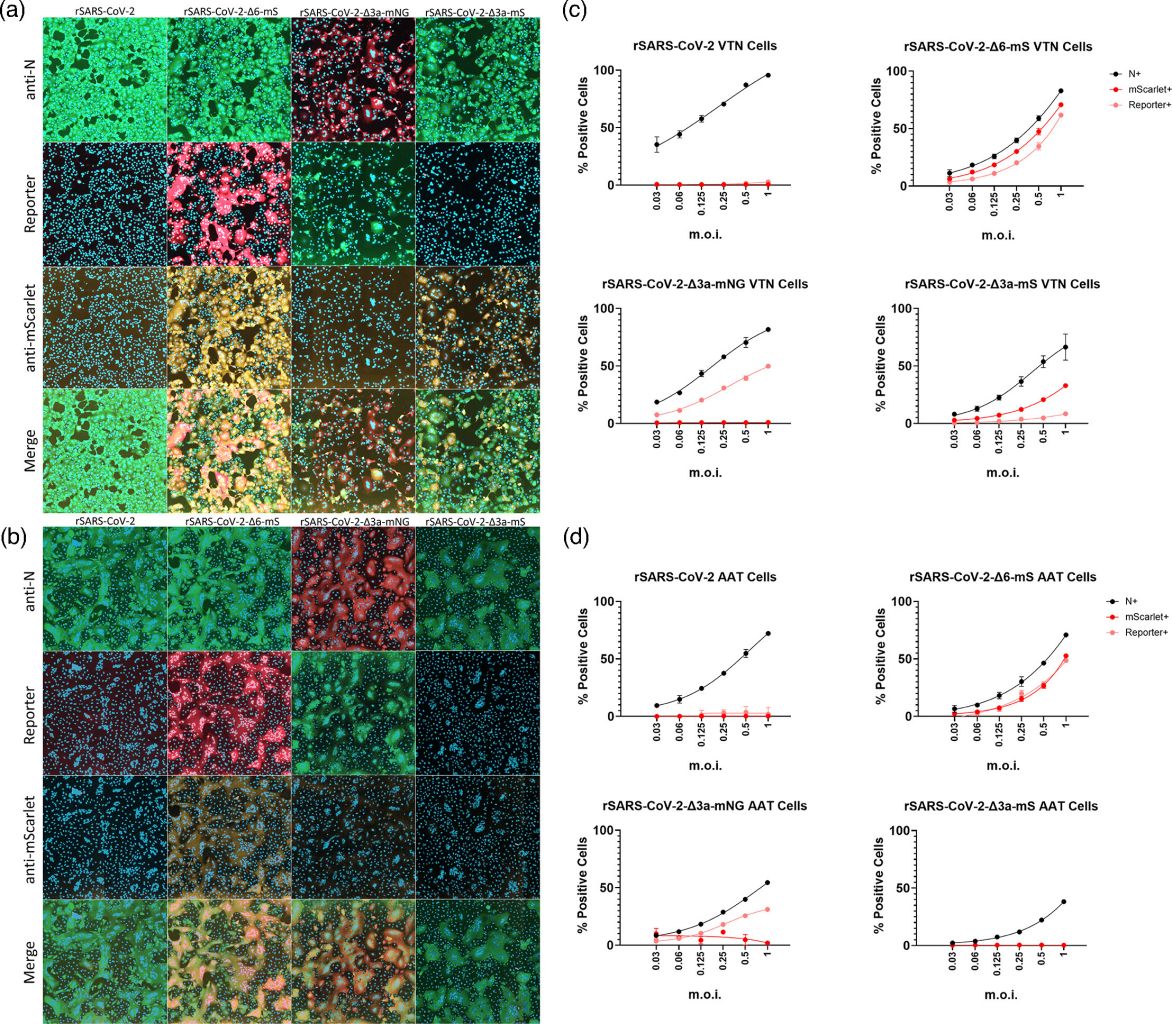
Detection of mS protein by IFA. Representative well images of immunostained (**a**) VTN or (**b**) AAT cells. VTN cells were infected with the indicated viruses at an m.o.i. of 1 (IU/cell) and fixed 18 hpi. Cells were then stained with anti-mS and anti-N antibodies. Nuclei were counterstained with Hoechst. Cell scoring analysis of (**c**) VTN or (**d**) AAT cells was performed on cells infected with the indicated range of m.o.i. values and fixed 18 hpi, based on the expression of either anti-N (N+)/ anti-mS (mScarlet+) detected by IFA or intrinsic reporter protein fluorescence [mS/mNG (Reporter+)]. Image analysis was performed using the CellReporterXpress software. Graphs show the mean and sem of *N*=3.

To investigate whether rSARS-CoV-2-Δ3a-mS expressed an mS protein that was either not fluorescent or fluorescent below the limit of detection of the assay, an immunostaining approach was employed for the detection of the mS protein. VTN and AAT cells were infected with rSARS-CoV-2, rSARS-CoV-2-Δ6-mS, rSARS-CoV-2-Δ3a-mNG and rSARS-CoV-2-Δ3a-mS at a range of m.o.i. values (Fig. 3). rSARS-CoV-2 and a rSARS-CoV-2 expressing mS in place of ORF6 (rSARS-CoV-2-Δ6-mS) served as negative and positive controls for the anti-mS antibody, respectively. At 18 hpi, the cells were fixed and immunostained with antibodies recognizing the N and mS proteins (Fig. 3a, b). Imaging analysis of the immunostained and fluorescent cells revealed that for both VTN and AAT cells, a robust intrinsic fluorescence was observed using rSARS-CoV-2-Δ6-mS and rSARS-CoV-2-Δ3a-mNG for infection, but not rSARS-CoV-2-Δ3a-mS (Fig. 3c, d). The mS fluorescence detected in rSARS-CoV-2-Δ6-mS infected cells recapitulated the values obtained by immunostaining against the N and mS proteins (Fig. 3c, d). However, the mS protein could only be detected in ~30–50% of the VTN cells infected with rSARS-CoV-2-Δ3a-mS (N-positive cells) by immunostaining, whereas for AAT cells, mS could not be detected by intrinsic fluorescence or immunostaining.

### Analysis of the viral transcriptomes by dRNAseq

To determine if insertion of the fluorophore coding sequences into that of ORF3a influenced the expression levels of the corresponding or adjacent sgRNAs, the viral transcriptomes of rSARS-CoV-2, rSARS-CoV-2-Δ3a-mNG and rSARS-CoV-2-Δ3a-mS in AAT cells at 18 hpi were analysed using dRNAseq and aligned to the corresponding genome sequences. SARS-CoV-2 sgRNA molecules were identified via the presence of a TRS using minimap2 and a pipeline previously developed at the University of Bristol [47,48]. The mapped sgRNAs correspond to the product associated with each TRS sequence. Therefore, mapped reads for the ORF3a TRS sgRNA in the rSARS-CoV-2-Δ3a-mNG and rSARS-CoV-2-Δ3a-mS correspond to transcripts of mNG or mS, respectively.

**Fig. 4.**
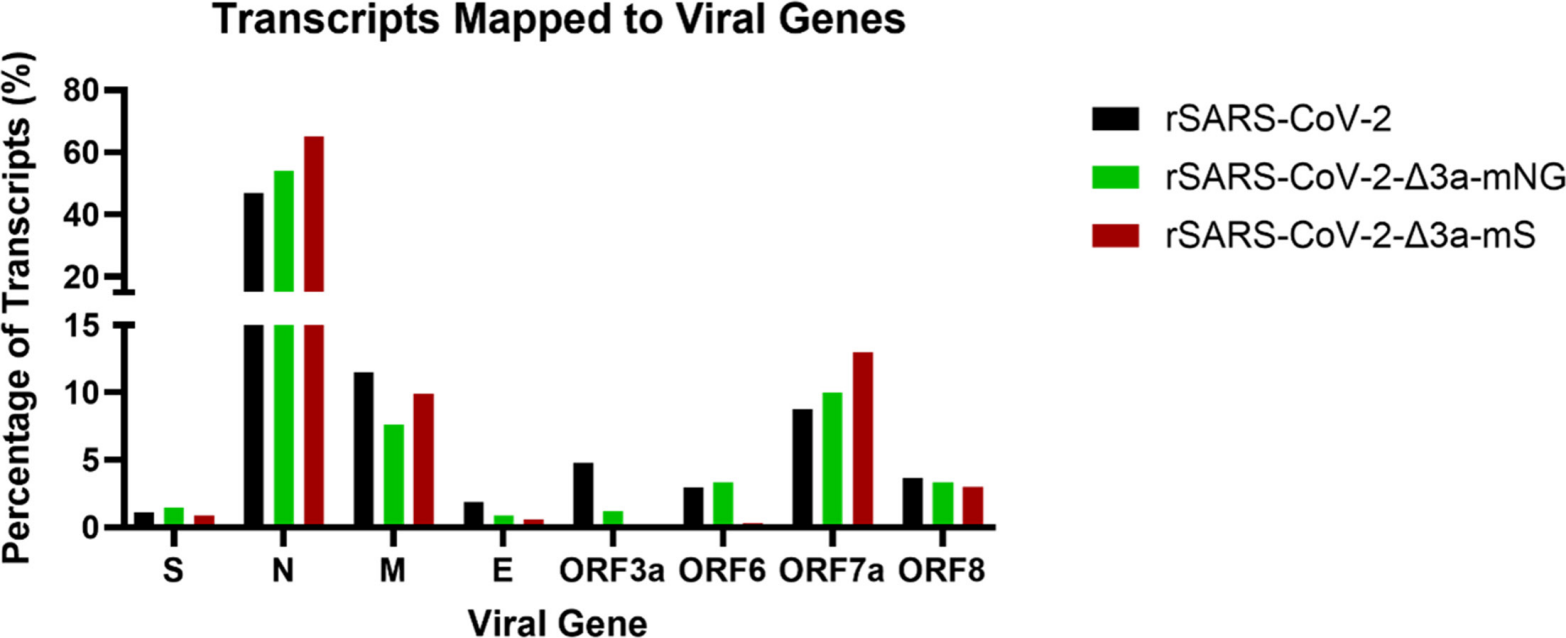
Analysis of rSARS-CoV-2-Δ3a-mNG and rSARS-CoV-2-Δ3a-mS sgRNA transcription in AAT cells. AAT cells were infected with rSARS-CoV-2, rSARS-CoV-2-Δ3a-mNG and rSARS-CoV-2-Δ3a-mS at an m.o.i. of 0.01 (IU/cell). At 18 hpi, cell lysates were harvested using TRIzol and RNA extracted. Viral RNA transcripts which mapped to the SARS-CoV-2 genome and possess a leader TRS sequence were identified, and the percentage of total transcripts was plotted for SARS-CoV-2 sgRNAs, as shown.

Transcript levels for eight SARS-CoV-2 sgRNAs were analysed which revealed that overall, the rSARS-CoV-2-Δ3a-mNG and rSARS-CoV-2-Δ3a-mS viruses produced similar relative viral sgRNA transcript levels as for the parental rSARS-CoV-2 except for the transcripts expressed from the ORF3a-TRS in rSARS-CoV-2-Δ3a-mNG/rSARS-CoV-2-Δ3a-mS-infected cells and the ORF6-TRS in rSARS-CoV-2-Δ3a-mS-infected cells (Fig. 4). The sgRNA associated with the ORF3a-TRS was reduced by ~80% in rSARS-CoV-2-Δ3a-mNG and absent in rSARS-CoV-2-Δ3a-mS-infected cells, respectively, compared to rSARS-CoV-2. Interestingly, whilst the transcript abundance of the ORF6 sgRNA was comparable between rSARS-CoV-2-Δ3a-mNG and rSARS-CoV-2-infected cells, it showed an ~90% reduction in rSARS-CoV-2-Δ3a-mS-infected cells (Fig. 4). The data suggested that rSARS-CoV-2-Δ3a-mNG but not rSARS-CoV-2-Δ3a-mS is able to transcribe sgRNAs from the ORF3a-TRS in AAT cells.

### rSARS-CoV-2-Δ3a-mS loses the mS insertion upon passage in VTN cells

**Fig. 5.**
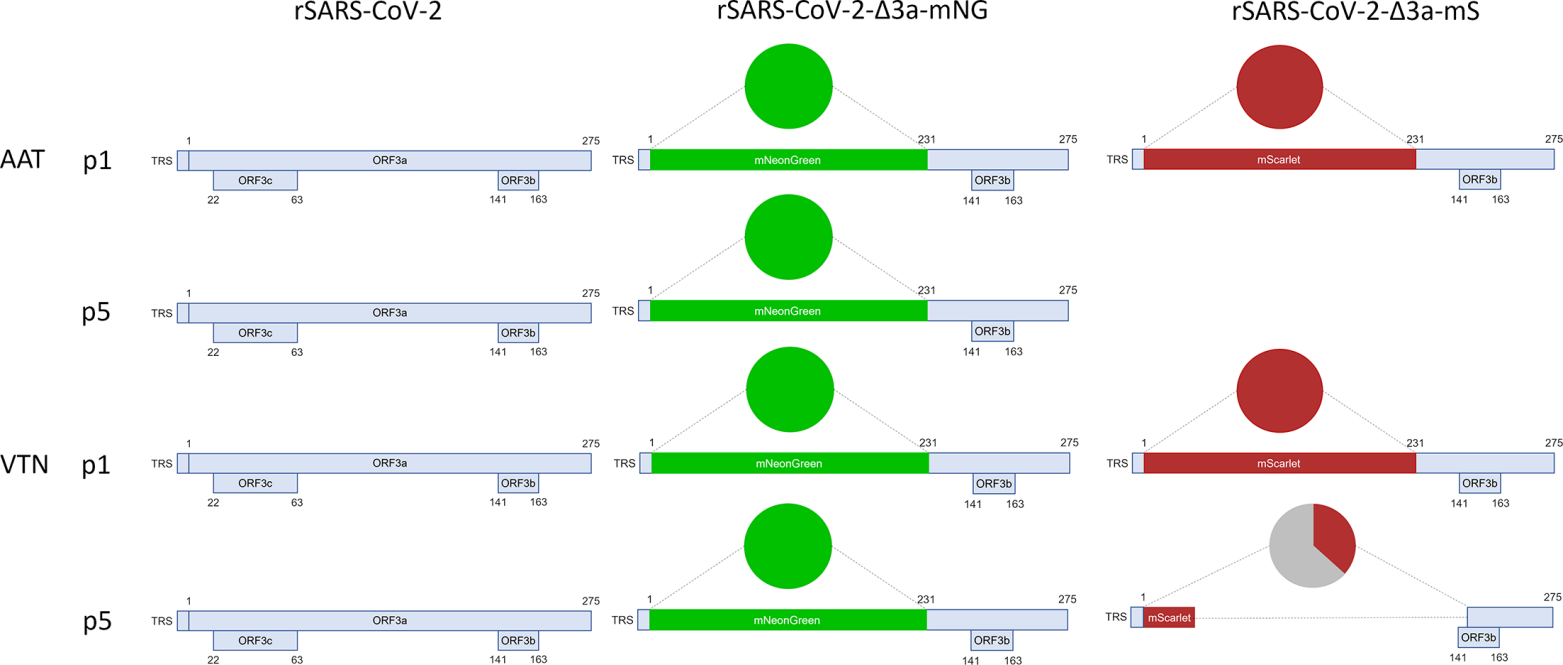
Selective pressure exists to remove the mScarlet insertion. The schematic represents the ORF3a coding sequence with or without each fluorescent reporter replacement. The locations of ORF3c and ORF3b are shown with the start and end aa (+1 frame) relative to ORF3a. The fluorescent reporter sequences are highlighted for rSARS-CoV-2-Δ3a-mNG and rSARS-CoV-2-Δ3a-mS in green and red, respectively. rSARS-CoV-2, rSARS-CoV-2-Δ3a-mNG and rSARS-CoV-2-Δ3a-mS were inoculated onto AAT or VTN cells at an m.o.i. of 0.01 (IU/cell). The supernatant was harvested at 72 hpi and used for passaging five times, diluted one in ten at each round. At p1 and p5, viral RNA in the culture supernatants was isolated, amplified over the ORF3a gene region by RT-PCR and Sanger sequenced. Three independent experiments were done. Circles represent pie charts showing the proportion of intact reporter protein sequence present in each biological repeat.

The partial replacement of the ORF3a coding sequence with that of a fluorescent reporter protein is a large modification to the viral genome. Consequently, the genetic stability of the rSARS-CoV-2-Δ3a-mNG and rSARS-CoV-2-Δ3a-mS viruses was examined over five sequential passages in VTN and AAT cells in three independent passaging experiments. rSARS-CoV-2 was included in the experiment as a control. At p1 and p5, viral RNA was extracted from the culture supernatants, and a 3197 bp region spanning the 3′ terminal 1292 nt of the S sequence to nt 88 of the ORF6 sequence (nt 24093–27289 relative to NC_045512) and encompassing the fluorophore sequences introduced into the ORF3a sequence was amplified by RT-PCR followed by Sanger sequencing (Fig. 5).

rSARS-CoV-2-Δ3a-mNG displayed greater genetic stability than rSARS-CoV-2-Δ3a-mS. After five passages of rSARS-CoV-2-Δ3a-mNG in both VTN and AAT cells, the mNG gene sequence and surrounding ORF3a sequence remained unchanged for all three repeats (Fig. 5). By contrast, rSARS-CoV-2-Δ3a-mS was not detectable by RT-PCR in the AAT cell culture supernatants after five passages and found to be genetically unstable in VTN cells, with a partial deletion of the mS coding sequence observed in two of three repeats by p5. The observed out-of-frame deletions were 632 (25545 – 26176) and 494 (del25579 – 26072) nt in length, with each of them leaving the ORF3-TRS and mS start codon intact with residual mS and ORF3a sequences downstream. Both deletions removed the central chromophore region of the mS protein; therefore, infection with these viruses would not generate a fluorescent signal. The two deletions resulted in ORFs encoding two novel putative gene products. The deletion of 632 nt retained mS codons 1–50 in-frame, with an additional two codons within the ORF3a sequence (mS_1-50_**-**AY*) before truncation. This deletion of ORF3a sequence also removed the first 24 nt of ORF3b including the start codon whilst retaining the last 382 nt of the ORF3a sequence. The deletion of 494 nt retained mS codons 1–69 in-frame, as well as an additional 21 codons (mS_1-69_**-**SKNNNEALALLEMPFQKPITL*) before truncation. Further analysis of the appearance of the 632 nt deletion, using viral RNA extracted from the infected VTN p2, p3 and p4 supernatants, showed that the deletion first appeared in p3 and was fully predominant by p5 (data not shown). A stock of the p5 virus (termed rSARS-CoV-2-Δ3a-ΔmS) was generated in VAT cells for further downstream analysis.

### Loss of ORF3a and presence of the mS both contribute to reduced viral replication *in vitro*

**Fig. 6.**
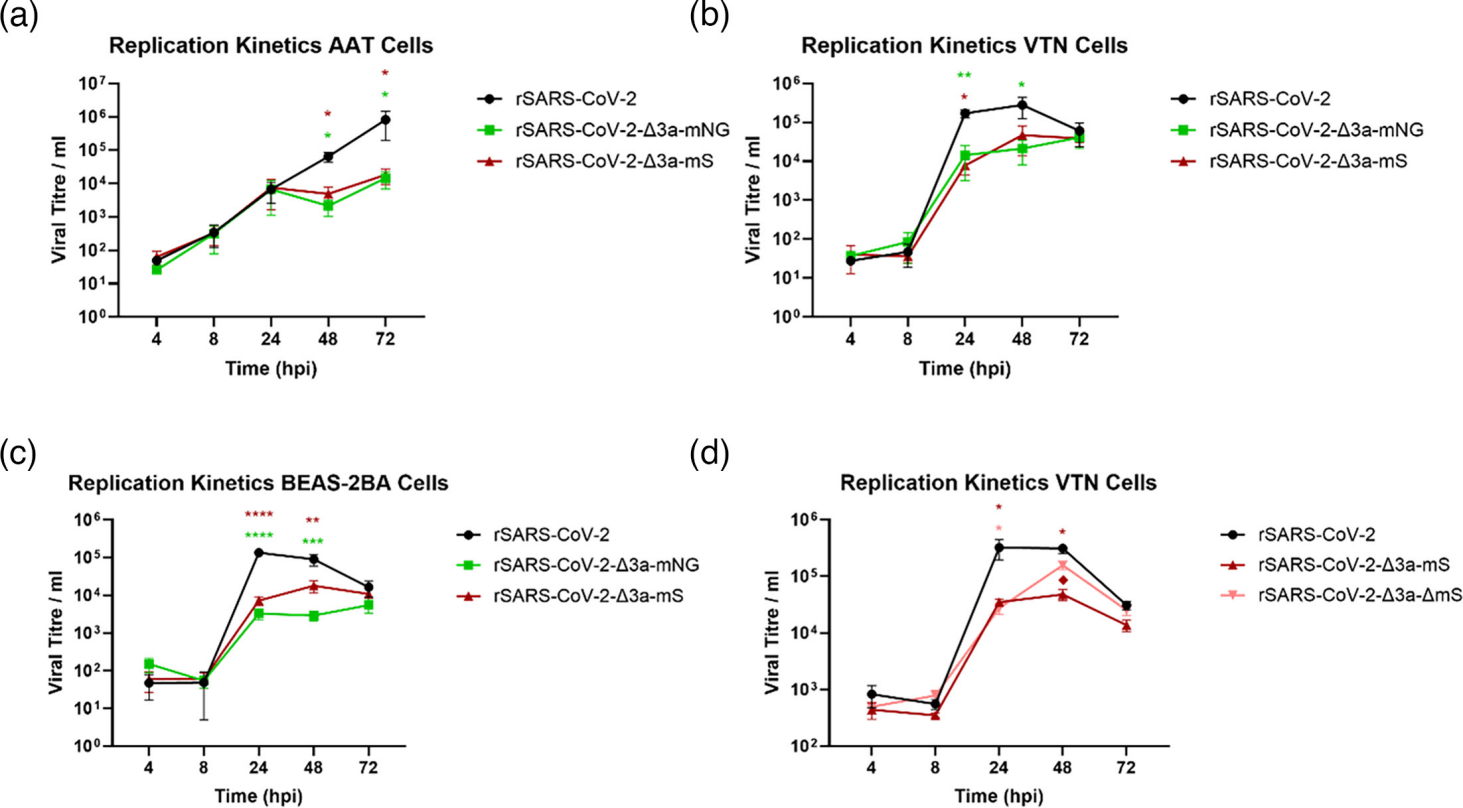
Replication of rSARS-CoV-2-Δ3a-mNG and rSARS-CoV-2-Δ3a-mS is attenuated compared to rSARS-CoV-2. (**a**) AAT, (**b**) VTN and (**c**) BEAS-2BA cells were infected with the rSARS-CoV-2, rSARS-CoV-2-Δ3a-mNG and rSARS-CoV-2-Δ3a-mS at an m.o.i. of 0.01 (IU/cell). (**d**) VTN cells were infected with rSARS-CoV-2, rSARS-CoV-2-Δ3a-mS and rSARS-CoV-2-Δ3a-ΔmS at an m.o.i. of 0.01. For all analyses, culture supernatants were sampled at 4, 8, 24, 48 and 72 hpi, and the viral titres in the supernatants were quantified by IFA analysis (targeting the N protein). Error bars represent ±sem of three independent experiments. Two-way ANOVA was performed, with significance taken at *P*-values <0.05, <0.005, 0.0005 and <0.0001 represented with *, **, *** and ****, respectively, in relation to rSARS-CoV-2 and in different colours corresponding to the different viruses. For (**d**), the significance between rSARS-CoV-2-ΔORF3a-mS and rSARS-CoV-2-ΔORF3a-ΔmS is represented with ♦.

The replication of rSARS-CoV-2-Δ3a-mNG and rSARS-CoV-2-Δ3a-mS compared to rSARS-CoV-2 was assessed in AAT, VTN and BEAS-2BA cells over a 72-h period to determine whether replacement of the ORF3a sequence with sequences encoding fluorescent reporter proteins affected viral growth (Fig. 6). BEAS-2BA cells were included in the comparison because, unlike AAT cells, which are tumour-derived alveolar cells, BEAS-2BA are immortalized, non-tumorigenic bronchial epithelial cells. In all cell lines assessed, the rSARS-CoV-2-Δ3a-mNG and rSARS-CoV-2-Δ3a-mS viruses were significantly attenuated in comparison with rSARS-CoV-2 at two of the assessed timepoints (Fig. 6a–c).

The 632 nt deletion in rSARS-CoV-2-Δ3a-ΔmS was detected in VTN cells. To assess whether deletion of the fluorescent reporter was advantageous for replication in VTN cells, the growth kinetics of rSARS-CoV-2, rSARS-CoV-2-Δ3a-mS and rSARS-CoV-2-Δ3a-ΔmS were compared. rSARS-CoV-2-Δ3a-ΔmS replicated comparably to rSARS-CoV-2-Δ3a-mS but to significantly lower levels than rSARS-CoV-2 at all timepoints except at 48 hpi when the titre of rSARS-CoV-2-Δ3a-ΔmS was similar to rSARS-CoV-2 (Fig. 6d). This increase in titre at 48 hpi suggested that the deletion of the mS coding sequence partially restored the rSARS-CoV-2-Δ3a-mS replication defect and that both the lack of the ORF3a sequence and the presence of the fluorescent reporter genes contributed to the reduced replication of the viruses.

### rSARS-CoV-2-Δ3a-mNG and rSARS-CoV-2-Δ3a-mS produce smaller plaques in comparison to rSARS-CoV-2, with moderate recovery in rSARS-CoV-2-Δ3a-ΔmS

**Fig. 7.**
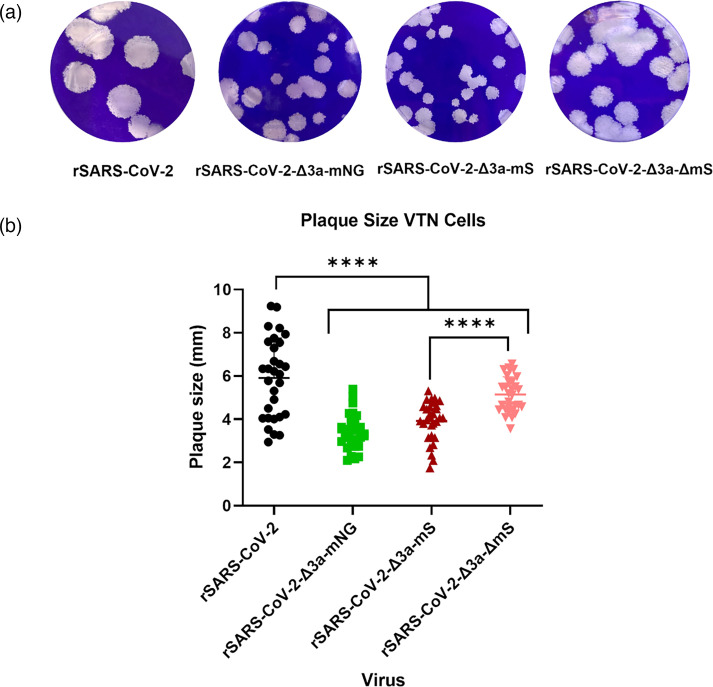
rSARS-CoV-2-Δ3a-mNG, rSARS-CoV-2-Δ3a-mS and rSARS-CoV-2-Δ3a-ΔmS produce smaller plaques than rSARS-CoV-2. (**a**) Representative images of plaques formed by rSARS-CoV-2, rSARS-CoV-2-Δ3a-mNG, rSARS-CoV-2-Δ3a-mS and rSARS-CoV-2-Δ3a-ΔmS. (**b**) Plaque diameter of 30 plaques per virus was measured using ImageJ software, over 3 biological repeats. Individual plaque diameters were plotted with error bars representing the sd. One-way ANOVA statistical analysis was performed, with *P*-values <0.0001 represented with ****.

To further investigate the attenuation in viral growth of rSARS-CoV-2-Δ3a-mNG, rSARS-CoV-2-Δ3a-mS and rSARS-CoV-2-Δ3a-ΔmS in cell culture, the plaque morphologies and sizes compared to rSARS-CoV-2 were assessed in VTN cells. The diameter of plaques produced by rSARS-CoV-2-Δ3a-mNG, rSARS-CoV-2-Δ3a-mS and rSARS-CoV-2-Δ3a-ΔmS was significantly smaller than for rSARS-CoV-2 (Fig. 7). Reflecting the growth curve analysis, the plaques produced by rSARS-CoV-2-Δ3a-ΔmS had an increased diameter in comparison to those produced by rSARS-CoV-2-Δ3a-mS, although they were still significantly smaller than plaques produced by rSARS-CoV-2 (Fig. 7b).

### rSARS-CoV-2-Δ3a-mNG and rSARS-CoV-2-Δ3a-mS have altered IFN sensitivity

**Fig. 8.**
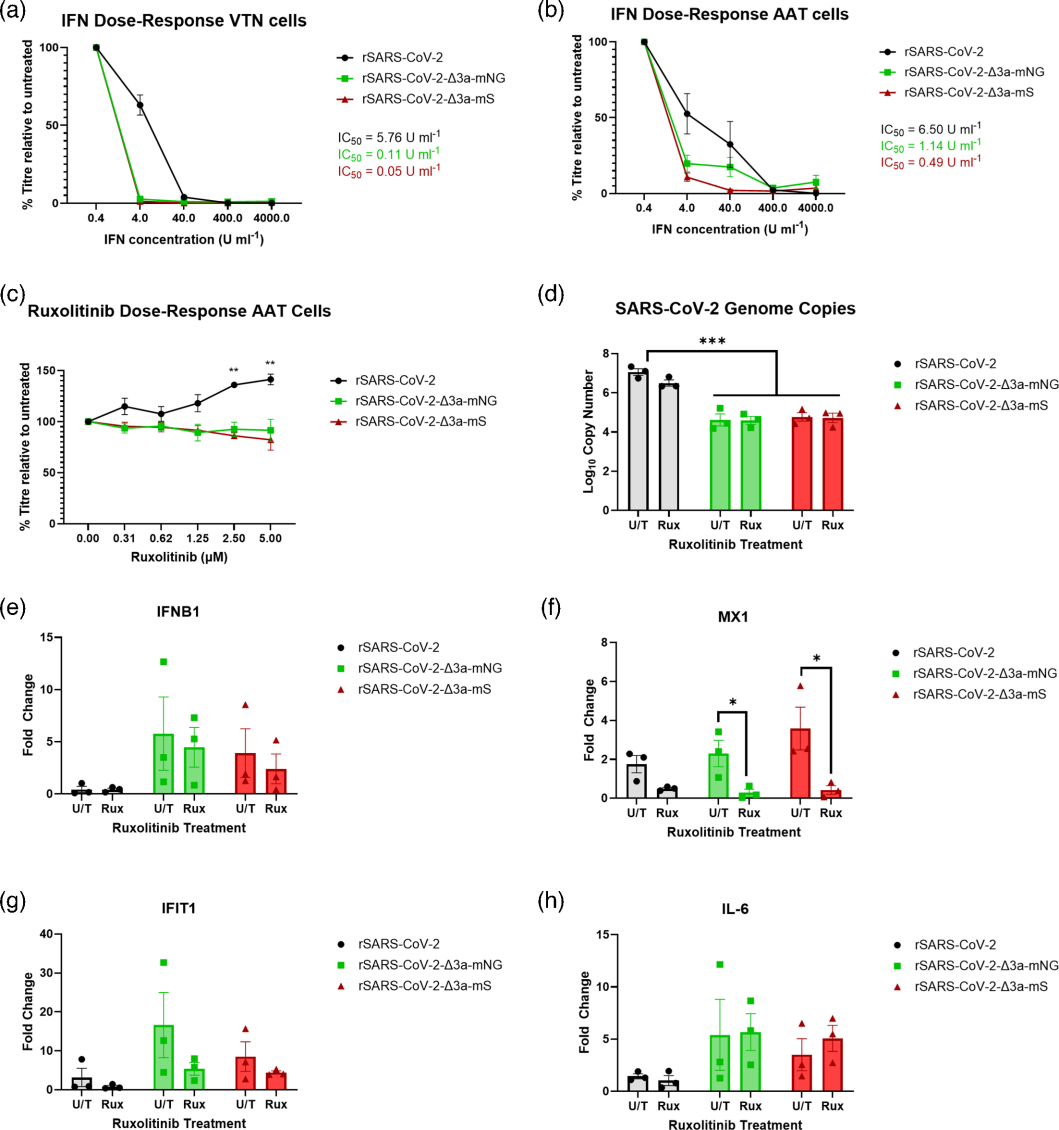
The effects of IFN-*α* and ruxolitinib on viral growth differ between rSARS-CoV-2, rSARS-CoV-2-Δ3a-mNG and rSARS-CoV-2-Δ3a-mS. (**a**) VTN and (**b**) AAT cells were pre-treated for 18 h with a range of IFN-*α* concentrations. Cells were then infected with rSARS-CoV-2, rSARS-CoV-2-Δ3a-mNG and rSARS-CoV-2-Δ3a-mS in the absence of IFN-*α* at an m.o.i. of 0.01 (IU/cell). Culture supernatants were harvested at either 24 hpi (VTN) or 48 hpi (AAT), and viral titres were determined by IFA titration and expressed as per cent titre of virus produced from cells untreated with IFN (per cent titre relative to untreated). (**c**) AAT cells were pre-treated with titrated amounts of ruxolitinib for 2 h. Cells were then infected at an m.o.i. of 0.025 and incubated with the indicated concentrations of ruxolitinib for 24 h. Relative infection was determined by IFA titration. (**d–h**) AAT cells were inoculated with the virus at an m.o.i. of 0.01. At 24 hpi, total RNA was extracted from the cells and used for RT-qPCR analysis of viral (**d**) and host gene transcript levels (**e–h**). Error bars represent ±sem of three independent experiments. Statistical analysis was performed using a two-way ANOVA with significance taken as *P*-values <0.05, <0.005 and <0.0005 represented with *, ** and ***, respectively.

The ORF3a protein has previously been shown to block the JAK-STAT signalling pathway [36,37]. To establish whether partial replacement of the ORF3a coding sequence with those of the fluorophores increased the IFN sensitivity of SARS-CoV-2, AAT and VTN cells were dosed with different amounts of IFN-*α* prior to infection with rSARS-CoV-2, rSARS-CoV-2-Δ3a-mNG and rSARS-CoV-2-Δ3a-mS. The cell supernatants were harvested at 24 and 48 hpi for VTN and AAT cells respectively, as dictated by the significantly different replication kinetics of the viruses in these cell types (Fig. 6). The IC_50_ values for rSARS-CoV-2-Δ3a-mNG and rSARS-CoV-2-Δ3a-mS were lower than that of rSARS-CoV-2 for both VTN and AAT cells (Fig. 8a, b). This suggested that the loss of the ORF3a coding sequence impaired the ability of rSARS-CoV-2 to tolerate the effects of IFN treatment.

To assess whether the absence of ORF3a contributed to the increased sensitivity of viral replication to IFN-*α* in AAT cells, these cells were treated with the JAK-STAT signalling inhibitor ruxolitinib during infection with rSARS-CoV-2, rSARS-CoV-2-Δ3a-mNG and rSARS-CoV-2-Δ3a-mS. Interestingly, unlike rSARS-CoV-2 which showed increased replication with increased concentrations of ruxolitinib, the replication of rSARS-CoV-2-Δ3a-mNG and rSARS-CoV-2-Δ3a-mS did not respond to ruxolitinib treatment as anticipated (Fig. 8c).

To confirm that ruxolitinib treatment effectively blocked JAK-STAT signalling, the transcript levels of selected innate immune genes were measured by RT-qPCR in virus-infected AAT cells, either treated with ruxolitinib or left untreated. Based on a previous study examining SARS-CoV-2-induced innate immune activation [50], the genes IFNB1, MX1, IFIT1 and IL-6 were selected for analysis. At 24 hpi, RT-qPCR revealed a significant decrease in viral transcript levels in cells infected with rSARS-CoV-2-Δ3a-mNG and rSARS-CoV-2-Δ3a-mS compared to rSARS-CoV-2, regardless of ruxolitinib treatment (Fig. 8d). This finding supported the attenuated replication phenotype observed in Fig. 6. Interestingly, the viruses lacking ORF3a showed a greater upregulation of IFNB1 gene transcription (Fig. 8e). The activity of ruxolitinib was confirmed by its suppression of the transcription of the IFN-sensitive genes (ISGs) MX1 and IFIT1. By contrast, IL-6 transcription, which was upregulated in response to virus infection but is not regulated by IFN, remained unaffected by ruxolitinib treatment, as expected (Fig. 8f–h).

## Discussion

The ORF3a accessory protein has been hypothesized to participate in almost all stages of the virus life cycle [23]. rSARS-CoV-2 viruses with fluorescent reporter genes inserted in place of viral genes are a valuable tool to investigate protein function in the context of virus infection. In this study, rSARS-CoV-2 viruses with a knockout of the ORF3a protein were successfully constructed, therefore showing that this protein is dispensable for viral replication *in vitro*, as corroborated by previous research on both SARS-CoV [19,56, 57] and SARS-CoV-2 [26,37, 58]. The results of previous studies reinforced the prominent role of ORF3a in viral pathogenesis and argued the benefit of incorporating an ORF3a gene deletion into potential rationally attenuated vaccine candidates [19,26, 37, 56, 58]. In this study, we took the novel approach of partially replacing the ORF3a coding sequence with those encoding fluorescent reporter proteins, abrogating the production of ORF3a, and assessed the resulting viruses as suitable reporter viruses.

Computational analyses have previously identified five ORFs within the ORF3a sgRNA that could potentially be translated into functional proteins [12] with ORF3a, ORF3b and ORF3c being the most extensively studied. Exogenous expression of these three ORFs in human cell lines has demonstrated their ability to antagonize different components of the IFN response. ORF3a has been shown to inhibit JAK-STAT signalling [36,37], whilst ORF3b [11] and ORF3c suppress IFN-*β* production. For ORF3c, this effect is mediated by the induction of MAVS degradation [53,59].

High-throughput proteomic [48,60], ribosomal profiling [61] and immunolocalization studies [23,59] have provided evidence for the production of the ORF3a and ORF3c proteins in SARS-CoV-2-infected cells. However, there is no evidence that the ORF3b protein is produced. Unlike ORF3c, which is proposed to be translated via a leaky scanning mechanism, the mechanism for ORF3b remains undefined. Translation of ORF3b via leaky scanning would require bypassing eight AUG codons, many of which are associated with moderate-to-strong Kozak sequences [62], making this mechanism unlikely. rSARS-CoV-2-Δ3a-mNG and rSARS-CoV-2-Δ3a-mS were constructed by replacing part of ORF3a and all of ORF3c with the mNG and mS coding sequences, respectively, whilst retaining the ORF3b coding sequence. However, both constructs retained multiple AUG codons (seven in rSARS-CoV-2-Δ3a-mNG and eight in rSARS-CoV-2-Δ3a-mS) upstream of ORF3b, each followed by in-frame stop codons, indicating that ORF3b is unlikely to be expressed in cells infected with these viruses.

Inserting the mNG or mS coding sequences in the ORF3a region of the genome did not result in the production of a fluorescent signal that was faithful to the number of infected (N positive) cells (Fig. 2c). This is likely due to the relative expression levels of the ORF3a and N proteins during SARS-CoV-2 infection. The analysis of the viral transcriptome in virus-infected AAT cells showed that transcripts expressed from the ORF3a-TRS in rSARS-CoV-2-Δ3a-mNG and rSARS-CoV-2-Δ3a-mS infected cells were decreased by ~90% or abolished respectively compared to rSARS-CoV-2-infected cells. A similar but less pronounced effect may have also occurred in VTN cells, diminishing the production of the fluorescent reporter proteins. In addition, the N gene, located at the extreme 3′ end of the genome, is the first sgRNA produced during discontinuous transcription and the most abundant viral protein in infected cells due to 3′ end bias. Therefore, quantifying infected cells using the N signal enables the detection of cells at earlier stages of infection compared to ORF3a.

SARS-CoV-2 viral genome replication and transcription takes place on double-membrane vesicles, to generate the sgRNAs [63,64]. It has previously been hypothesized that ORF3a is involved in the formation of these structures [23]. The levels of viral transcripts produced in rSARS-CoV-2-Δ3a-mNG-infected cells were largely comparable to the parental rSARS-CoV-2 with the exception of transcripts using the ORF3a-TRS and the ORF6-TRS (for rSARS-CoV-2-Δ3a-mS only) (Fig. 4). This indicates that the deleterious effect on viral replication observed in Fig. 6a–c was unlikely due to aberrant transcription of all the SARS-CoV-2 sgRNAs produced.

Notably, rSARS-CoV-2-Δ3a-mS did not produce a robust fluorescent signal (Fig. 2). In VTN but not AAT cells, the mS protein could be detected by immunostaining (Fig. 3c, d). Furthermore, viral transcriptomic analysis of infected AAT cells did not result in the detection of transcripts generated by either the ORF3a-TRS or ORF6-TRS for the rSARS-CoV-2-Δ3a-mS virus (Fig. 4). Taken together, the results suggest that the rSARS-CoV-2-Δ3a-mS virus is unable to produce the mS reporter protein in AAT cells.

The mNG and mS coding sequences are large, and therefore, inserting them into the viral genome is a considerable modification to the genomic RNA structure. Interestingly, the rSARS-CoV-2-Δ3a-mNG virus was found to be genetically stable in both AAT and VTN cells unlike the rSARS-CoV-2-Δ3a-mS virus (Fig. 5). This virus acquired deletions of the mS gene sequence in two-thirds of the replicates in VTN cells, showing a strong selective pressure to remove the mS coding sequence, creating the virus rSARS-CoV-2-Δ3a-ΔmS (Fig. 5). Interestingly, in AAT cells, the rSARS-CoV-2-Δ3a-mS virus was not detectable by PCR at passage 5. This suggests that there is a cell-type-dependent tolerance to this insertion. The highly structured region overlapping the S and ORF3a genes has been proposed to adopt cell-type-specific RNA structures [65]. This cell-type-dependent RNA structure could be a potential explanation of the cell-type-dependent failure of rSARS-CoV-2-Δ3a-mS to produce detectable levels of an mS sgRNA from the ORF3a-TRS (Fig. 4). Additionally, partial deletions of mS were observed during passage in VTN cells (Fig. 5), which removed the ORF3b start codon and partially recovered the replication (Fig. 6) and plaque (Fig. 7) phenotype of the rSARS-CoV-2-Δ3a-mS virus. However, it was unlikely that these effects were due to the failure to produce ORF3b in infected cells but rather that the RNA structure of the mNG coding sequence, in the context of the ORF3a insertion, was less detrimental to viral replication than that of mS.

Previous studies evaluating the effects of ORF3a deletions on the phenotypes of SARS-CoV [19,56] and SARS-CoV-2 [26,37, 58] *in vitro* and *in vivo* have reported varying results. For SARS-CoV, the deletion of ORF3a whilst retaining ORF3b moderately reduced viral replication *in vitro* for recombinant viruses derived from both human and mouse-adapted strains [19,56]. However, *in vivo* studies using BALB/c mice yielded mixed results. Whilst no attenuation was observed for the human virus with an ORF3a deletion [56], the deletion of ORF3a from the mouse-adapted virus attenuated replication and reduced virulence [19].

For SARS-CoV-2, the deletion of ORF3a (including ORF3c and ORF3b) caused attenuated replication *in vivo* in the K18-hACE2 mouse model but not *in vitro*, although a small plaque phenotype was observed [26]. Additional deletion of ORF6, 7a or 7b further attenuated the virus, leading to 10- to 100-fold reductions in replication in Vero-E6 and human A549-hACE2 cells, as well as in K18-hACE2 mouse and hamster pathogenesis models [58]. In a different study, recombinant SARS-CoV-2 with deletions of ORFs 6, 7 and 8 (Δ678) was not significantly attenuated in cell culture. However, the additional deletion of the entire ORF3a coding sequence (Δ3678) resulted in mild attenuation in Vero-E6 cells and more significant attenuation in immune-competent Calu-3 and human airway epithelial (HAE) cells [59]. Examination of the individual ORF deletions in recombinant viruses derived from a mouse-adapted SARS-CoV-2 strain provided further insight into the roles of the individual ORFs. The deletion of ORF3a, but not ORF3b, reduced viral replication in the lungs of BALB/c mice. Similarly, to the Δ3678 virus, the ORF3a-deleted virus showed ~10-fold attenuation in Vero-E6 cells and stronger attenuation in Calu-3 and HAE cells [37]. In another study, specific mutations preserving ORF3a whilst preventing ORF3c translation revealed that the loss of ORF3c expression did not affect virus replication in cell culture or hamster tissues [59].

These studies highlight that the extent of the ORF3a deletion, the genetic background of the virus and the deletion of additional accessory ORFs can all contribute to the replication phenotypes observed *in vitro* and *in vivo*. In this study, rSARS-CoV-2-Δ3a-mS and rSARS-CoV-2-Δ3a-mNG both exhibited attenuated replication phenotypes in different cell lines (Fig. 6a–c) and reduced plaque size (Fig. 7b). Whilst this attenuation may be attributed to the loss of ORF3a, the insertion of mNG and mS into the SARS-CoV-2 genome was observed to alter transcript levels from the ORF3-TRS and, in the case of mS, from the ORF6-TRS, which may have contributed to further attenuation. Interestingly, deletion of the mS gene sequence partially restored the replication (Fig. 6d) and plaque size (Fig. 7b) of rSARS-CoV-2-Δ3a-mS. Thus, both the ORF3a deletion and the insertion of the fluorescent mS coding sequence played roles in the observed attenuated replication phenotypes.

A recognized role of the SARS-CoV-2 accessory proteins is the modulation of the host innate immune response [66]. The ORF3a accessory protein elicits these effects through several means, such as promoting the NLRP3 inflammasomal pathway by triggering the overexpression of cellular cytokines [35] whilst simultaneously blocking IFN signalling through the JAK-STAT pathway [36,37]. To validate whether the rSARS-CoV-2-Δ3a-mS and rSARS-CoV-2-Δ3a-mNG viruses could be used to investigate the role of ORF3a, the already established innate immune modulatory effects of ORF3a were assessed. The rSARS-CoV-2-Δ3a-mS and rSARS-CoV-2-Δ3a-mNG viruses were found to be more sensitive to IFN pre-treatment, in both AAT and VTN cells (Fig. 8a, b). This effect was more pronounced in the VTN cells as they are incapable of producing IFNs but are able to respond [67]. Additionally, unexpectedly, the replication of rSARS-CoV-2-Δ3a-mS and rSARS-CoV-2-Δ3a-mNG was not rescued by the inhibition of JAK-STAT signalling using ruxolitinib, unlike rSARS-CoV-2 (Fig. 8c).

We therefore examined the transcription profiles of the IFNB1, MX1, IFIT1 and IL-6 genes in virus-infected cells, both treated with ruxolitinib and left untreated. Similar to the findings of Liu *et al*. [37], who investigated the effects of ORF3a deletion on ISG expression in A549-hACE2 cells, we observed a significant decrease in viral transcripts in cells infected with viruses containing ORF3a deletions compared to parental viruses. However, there was an associated increase in ISG transcript levels. In this study, ruxolitinib treatment reduced the upregulation of MX1 and IFIT1 in cells infected with rSARS-CoV-2-Δ3a-mS and rSARS-CoV-2-Δ3a-mNG compared to rSARS-CoV-2 (Fig. 8f, g), confirming the expected suppression of ISG expression. Despite this suppression, there was no corresponding increase in intracellular viral replication levels, as determined by RT-qPCR. This indicates that the growth defects observed in rSARS-CoV-2-Δ3a-mS and rSARS-CoV-2-Δ3a-mNG-infected BEAS-2B and AAT cells were not due to the viruses’ inability to suppress JAK-STAT signalling in the absence of ORF3a. Furthermore, consistent with previous observations [37], rSARS-CoV-2-Δ3a-mS and rSARS-CoV-2-Δ3a-mNG were attenuated in Vero-E6 cells, which are deficient in IFN production. There are increasing reports of non-canonical, IFN-independent mechanisms driving ISG induction [68]. It is tempting to speculate that ORF3a may play a role in antagonizing one or more of these responses. The data from this study suggest that the viruses generated here are valuable tools for further exploring the role of ORF3a in host–virus interactions. Specifically, the viruses were shown to have a reduced tolerance to the host innate immune response, which does not appear to be reliant on JAK-STAT signalling.

Differing genetic stability (Fig. 5), transcriptional profiles (Fig. 3) and fluorescent reporter expression (Figs2  4) were observed between rSARS-CoV-2-Δ3a-mNG and rSARS-CoV-2-Δ3a-mS. Additionally, for rSARS-CoV-2-Δ3a-mS, different results were obtained when using either AAT or VTN cells (Figs2, 3, 4  5). Ultimately, we observed a cell-type-dependent failure of rSARS-CoV-2-Δ3a-mS to express the gene product of the ORF3a-TRS sgRNA transcript. These results could be explained either by a failure to translate the sgRNA, a critically low abundance of the sgRNA or a failure to utilize the ORF3a-TRS during infection of AAT cells.

Fluorescent reporter viruses are useful tools to allow for high-throughput analysis of antiviral compound screening. Faithful recapitulation of results generated with WT virus is necessary to allow for trustworthy data to be obtained using fluorescent reporter viruses. Replacement of the ORF3a sequence with either mS or mNG has not produced useful reporter viruses due to an attenuated replication phenotype and genetic instability. Additionally, due to the slight recovery of the attenuated phenotype upon deletion of the mS sequence, it appears that both insertion of the fluorescent reporter gene and deletion of the ORF3a sequence were responsible for the attenuation of the viruses. We hypothesize that this is due to the differing RNA structures of the two reporter genes. Therefore, although these viruses can be used to analyse the effect of ORF3a knockout on viral replication, the additional impact of the reporter gene insertion must be considered. In conclusion, the replacement of the ORF3a sequence with fluorescent reporters is not an ideal strategy for the generation of fluorescent reporter viruses.

## Abbreviations

ACE2: angiotensin-converting enzyme 2
CPE: cytopathic effect
dRNAseq: direct RNA sequencing
hpi: h post-infection
IFA: immunofluorescence assay
ISG: IFN-sensitive gene
IU/ml: infectious units per millilitre
IVT: *in vitro* transcription
JAK: Janus kinase
LB: lysogeny broth
MEM: minimal essential medium
p1: passage 1
PBM: PDZ-binding motif
PFA: paraformaldehyde
RFP: red fluorescent protein
RTC: replication–transcription complex
RT-PCR: reverse transcriptase-polymerase chain reaction
RT-qPCR: real-time quantitative PCR
SARS-CoV-2: Severe acute respiratory syndrome coronavirus 2
sgRNAs: sub-genomic RNAs
STAT: signal transducer and activator of transcription
TAR: transformation-associated recombination
TRS: transcriptional regulatory sequence

## References

[1] Zhou P, Yang XL, Wang XG, Hu B, Zhang L (2020). A pneumonia outbreak associated with a new coronavirus of probable bat origin. Nature.

[2] Wu F, Zhao S, Yu B, Chen YM, Wang W (2020). A new coronavirus associated with human respiratory disease in China. Nature.

[3] Wu P, Hao X, Lau EHY, Wong JY, Leung KSM (2020). Real-time tentative assessment of the epidemiological characteristics of novel coronavirus infections in Wuhan, China, as at 22 January 2020. Euro Surveill.

[4] Kesheh MM, Hosseini P, Soltani S, Zandi M (2022). An overview on the seven pathogenic human coronaviruses. Rev Med Virol.

[5] Wu A, Peng Y, Huang B, Ding X, Wang X (2020). Genome composition and divergence of the novel coronavirus (2019-nCoV) originating in China. Cell Host Microbe.

[6] Ziebuhr J (2005). The coronavirus replicase. Curr Top Microbiol Immunol.

[7] Sawicki SG, Sawicki DL, Siddell SG (2007). A contemporary view of coronavirus transcription. J Virol.

[8] Shang J, Han N, Chen Z, Peng Y, Li L (2021). Compositional diversity and evolutionary pattern of coronavirus accessory proteins. *Brief Bioinform*.

[9] Marra MA, Jones SJM, Astell CR, Holt RA, Brooks-Wilson A (2003). The genome sequence of the SARS-associated coronavirus. Science.

[10] Yu C-J, Chen Y-C, Hsiao C-H, Kuo T-C, Chang SC (2004). Identification of a novel protein 3a from severe acute respiratory syndrome coronavirus. FEBS Lett.

[11] Konno Y, Kimura I, Uriu K, Fukushi M, Irie T (2020). SARS-CoV-2 ORF3b is a potent interferon antagonist whose activity is increased by a naturally occurring elongation variant. Cell Rep.

[12] Jungreis I, Nelson CW, Ardern Z, Finkel Y, Krogan NJ (2021). Conflicting and ambiguous names of overlapping ORFs in the SARS-CoV-2 genome: a homology-based resolution. Virology.

[13] Firth AE (2020). A putative new SARS-CoV protein, 3c, encoded in an ORF overlapping ORF3a. J Gen Virol.

[14] Lu W, Zheng B-J, Xu K, Schwarz W, Du L (2006). Severe acute respiratory syndrome-associated coronavirus 3a protein forms an ion channel and modulates virus release. Proc Natl Acad Sci USA.

[15] Schwarz S, Wang K, Yu W, Sun B, Schwarz W (2011). Emodin inhibits current through SARS-associated coronavirus 3a protein. Antiviral Res.

[16] Kern DM, Sorum B, Mali SS, Hoel CM, Sridharan S (2021). Cryo-EM structure of SARS-CoV-2 ORF3a in lipid nanodiscs. Nat Struct Mol Biol.

[17] Miller AN, Houlihan PR, Matamala E, Cabezas-Bratesco D, Lee GY (2023). The SARS-CoV-2 accessory protein Orf3a is not an ion channel, but does interact with trafficking proteins. Elife.

[18] Caillet-Saguy C, Durbesson F, Rezelj VV, Gogl G, Tran QD (2021). Host PDZ-containing proteins targeted by SARS-CoV-2. FEBS J.

[19] Castaño-Rodriguez C, Honrubia JM, Gutiérrez-Álvarez J, DeDiego ML, Nieto-Torres JL (2018). Role of severe acute respiratory syndrome coronavirus viroporins E, 3a, and 8a in replication and pathogenesis. mBio.

[20] Piserchio A, Spaller M, Mierke DF (2006). Targeting the PDZ domains of molecular scaffolds of transmembrane ion channels. AAPS J.

[21] Luck K, Charbonnier S, Travé G (2012). The emerging contribution of sequence context to the specificity of protein interactions mediated by PDZ domains. FEBS Lett.

[22] Chen D, Zheng Q, Sun L, Ji M, Li Y (2021). ORF3a of SARS-CoV-2 promotes lysosomal exocytosis-mediated viral egress. Dev Cell.

[23] Zhang J, Ejikemeuwa A, Gerzanich V, Nasr M, Tang Q (2022). Understanding the role of SARS-CoV-2 ORF3a in viral pathogenesis and COVID-19. Front Microbiol.

[24] Wong SLA, Chen Y, Chan CM, Chan CSM, Chan PKS (2005). In *vivo* functional characterization of the SARS-Coronavirus 3a protein in *Drosophila*. Biochem Biophys Res Commun.

[25] Zhang Y, Sun H, Pei R, Mao B, Zhao Z (2021). The SARS-CoV-2 protein ORF3a inhibits fusion of autophagosomes with lysosomes. Cell Discov.

[26] Silvas JA, Vasquez DM, Park J-G, Chiem K, Allué-Guardia A (2021). Contribution of SARS-CoV-2 accessory proteins to viral pathogenicity in K18 human ACE2 transgenic mice. J Virol.

[27] Del Valle DM, Kim-Schulze S, Huang H-H, Beckmann ND, Nirenberg S (2020). An inflammatory cytokine signature predicts COVID-19 severity and survival. Nat Med.

[28] González-Navajas JM, Lee J, David M, Raz E (2012). Immunomodulatory functions of type I interferons. Nat Rev Immunol.

[29] Darnell JE, Kerr IM, Stark GR (1994). Jak-STAT pathways and transcriptional activation in response to IFNs and other extracellular signaling proteins. Science.

[30] Lei X, Dong X, Ma R, Wang W, Xiao X (2020). Activation and evasion of type I interferon responses by SARS-CoV-2. Nat Commun.

[31] Yuen C-K, Lam J-Y, Wong W-M, Mak L-F, Wang X (2020). SARS-CoV-2 nsp13, nsp14, nsp15 and orf6 function as potent interferon antagonists. Emerg Microbes Infect.

[32] Chen DY, Khan N, Close BJ, Goel RK, Blum B (2021). SARS-CoV-2 disrupts proximal elements in the JAK-STAT pathway. J Virol.

[33] Xia H, Cao Z, Xie X, Zhang X, Chen JY-C (2020). Evasion of type I interferon by SARS-CoV-2. Cell Rep.

[34] Zhang J, Li Q, Cruz Cosme RS, Gerzanich V, Tang Q (2021). Genome-wide characterization of SARS-CoV-2 cytopathogenic proteins in the search of antiviral targets. mBio.

[35] Xu H, Akinyemi IA, Chitre SA, Loeb JC, Lednicky JA (2022). SARS-CoV-2 viroporin encoded by ORF3a triggers the NLRP3 inflammatory pathway. Virology.

[36] Wang R, Yang X, Chang M, Xue Z, Wang W (2021). ORF3a protein of severe acute respiratory syndrome coronavirus 2 inhibits interferon-activated Janus kinase/signal transducer and activator of transcription signaling via elevating suppressor of cytokine signaling 1. Front Microbiol.

[37] Liu Y, Zhang X, Liu J, Xia H, Zou J (2022). A live-attenuated SARS-CoV-2 vaccine candidate with accessory protein deletions. Nat Commun.

[38] Chiem K, Morales Vasquez D, Park JG, Platt RN, Anderson T (2021). Generation and characterization of recombinant SARS-CoV-2 expressing reporter genes. J Virol.

[39] Thi Nhu Thao T, Labroussaa F, Ebert N, V’kovski P, Stalder H (2020). Rapid reconstruction of SARS-CoV-2 using a synthetic genomics platform. Nature.

[40] Fahnøe U, Pham LV, Fernandez-Antunez C, Costa R, Rivera-Rangel LR (2022). Versatile SARS-CoV-2 reverse-genetics systems for the study of antiviral resistance and replication. Viruses.

[41] Herrmann A, Jungnickl D, Cordsmeier A, Peter AS, Überla K (2021). Cloning of a passage-free SARS-CoV-2 genome and mutagenesis using red recombination. Int J Mol Sci.

[42] Bindels DS, Haarbosch L, van Weeren L, Postma M, Wiese KE (2017). mScarlet: a bright monomeric red fluorescent protein for cellular imaging. Nat Methods.

[43] Shaner NC, Lambert GG, Chammas A, Ni Y, Cranfill PJ (2013). A bright monomeric green fluorescent protein derived from *Branchiostoma lanceolatum*. Nat Methods.

[44] Rihn SJ, Merits A, Bakshi S, Turnbull ML, Wickenhagen A (2021). A plasmid DNA-launched SARS-CoV-2 reverse genetics system and coronavirus toolkit for COVID-19 research. PLoS Biol.

[45] Erdmann M, Williamson MK, Jearanaiwitayakul T, Bazire J, Matthews DA (2022). Development of SARS-CoV-2 replicons for the ancestral virus and variant of concern Delta for antiviral screening. *Microbiology*.

[46] Glitscher M, Spannaus IM, Behr F, Murra RO, Woytinek K (2024). The protease domain in HEV pORF1 mediates the replicase’s localization to multivesicular bodies and its exosomal release. Cell Mol Gastroenterol Hepatol.

[47] Donovan-Banfield I, Turnell AS, Hiscox JA, Leppard KN, Matthews DA (2020). Deep splicing plasticity of the human adenovirus type 5 transcriptome drives virus evolution. *Commun Biol*.

[48] Davidson AD, Williamson MK, Lewis S, Shoemark D, Carroll MW (2020). Characterisation of the transcriptome and proteome of SARS-CoV-2 reveals a cell passage induced in-frame deletion of the furin-like cleavage site from the spike glycoprotein. Genome Med.

[49] Schneider CA, Rasband WS, Eliceiri KW (2012). NIH Image to ImageJ: 25 years of image analysis. Nat Methods.

[50] Thorne LG, Reuschl AK, Zuliani-Alvarez L, Whelan MVX, Turner J (2021). SARS-CoV-2 sensing by RIG-I and MDA5 links epithelial infection to macrophage inflammation. EMBO J.

[51] Livak KJ, Schmittgen TD (2001). Analysis of relative gene expression data using real-time quantitative PCR and the 2(-Delta Delta C(T)) Method. Methods.

[52] Toelzer C, Gupta K, Yadav SKN, Borucu U, Davidson AD (2020). Free fatty acid binding pocket in the locked structure of SARS-CoV-2 spike protein. Science.

[53] Stewart H, Lu Y, O’Keefe S, Valpadashi A, Cruz-Zaragoza LD (2023). The SARS-CoV-2 protein ORF3c is a mitochondrial modulator of innate immunity. iScience.

[54] Yang H-C, Chen C-H, Wang J-H, Liao H-C, Yang C-T (2020). Analysis of genomic distributions of SARS-CoV-2 reveals a dominant strain type with strong allelic associations. Proc Natl Acad Sci U S A.

[55] Hou YJ, Chiba S, Halfmann P, Ehre C, Kuroda M (2020). SARS-CoV-2 D614G variant exhibits efficient replication ex *vivo* and transmission in *vivo*. Science.

[56] Yount B, Roberts RS, Sims AC, Deming D, Frieman MB (2005). Severe acute respiratory syndrome coronavirus group-specific open reading frames encode nonessential functions for replication in cell cultures and mice. J Virol.

[57] Freundt EC, Yu L, Goldsmith CS, Welsh S, Cheng A (2010). The open reading frame 3a protein of severe acute respiratory syndrome-associated coronavirus promotes membrane rearrangement and cell death. J Virol.

[58] Ye C, Park JG, Chiem K, Dravid P, Allué-Guardia A (2023). Immunization with recombinant accessory protein-deficient SARS-CoV-2 protects against lethal challenge and viral transmission. Microbiol Spectr.

[59] Müller M, Herrmann A, Fujita S, Uriu K, Kruth C (2023). ORF3c is expressed in SARS-CoV-2-infected cells and inhibits innate sensing by targeting MAVS. EMBO Rep.

[60] Bojkova D, Klann K, Koch B, Widera M, Krause D (2020). Proteomics of SARS-CoV-2-infected host cells reveals therapy targets. Nature.

[61] Finkel Y, Mizrahi O, Nachshon A, Weingarten-Gabbay S, Morgenstern D (2021). The coding capacity of SARS-CoV-2. Nature.

[62] Jungreis I, Sealfon R, Kellis M (2021). SARS-CoV-2 gene content and COVID-19 mutation impact by comparing 44 Sarbecovirus genomes. Nat Commun.

[63] Snijder EJ, Limpens RWAL, de Wilde AH, de Jong AWM, Zevenhoven-Dobbe JC (2020). A unifying structural and functional model of the coronavirus replication organelle: Tracking down RNA synthesis. PLoS Biol.

[64] Eymieux S, Rouillé Y, Terrier O, Seron K, Blanchard E (2021). Ultrastructural modifications induced by SARS-CoV-2 in vero cells: a kinetic analysis of viral factory formation, viral particle morphogenesis and virion release. Cell Mol Life Sci.

[65] Lan TCT, Allan MF, Malsick LE, Woo JZ, Zhu C (2022). Secondary structural ensembles of the SARS-CoV-2 RNA genome in infected cells. Nat Commun.

[66] Minkoff JM, tenOever B (2023). Innate immune evasion strategies of SARS-CoV-2. Nat Rev Microbiol.

[67] Desmyter J, Melnick JL, Rawls WE (1968). Defectiveness of interferon production and of rubella virus interference in a line of African green monkey kidney cells (Vero). J Virol.

[68] Swaraj S, Tripathi S (2024). Interference without interferon: interferon-independent induction of interferon-stimulated genes and its role in cellular innate immunity. mBio.

